# Supplementary material for: Criterion-Related Validity of Field-Based Fitness Tests in Adults: A Systematic Review
Source: J Clin Med. 2021 Aug 23;10(16):3743. doi: 10.3390/jcm10163743 (PMC8397016; doi:10.3390/jcm10163743)
Supplement: Supplementary file 1 [file jcm-10-03743-s001.zip › jcm-1272580 Supplementary.pdf]

Supplementary table S1. Quality assessment criteria for criterion-related validity studies.

Grading system parameter	Grade	Criterion
Number of study subjects	0	n ≤ 10
	1	n = 11-50
	2	n ≥ 51
Description of the study population with respect to age, sex, health status, fitness levels, pubertal status, ethnicity, physical activity patterns, body composition, etc.	0	Less items than required for grade 1
	1	At least age, sex, health status, and fitness levels
	2	More items than required for grade 1
Statistical analysis included in the study	0	Those not included in 1
	1	Error indexes or regression analysis
	2	≥ 3 items or Bland-Altman plot and/or ANOVA for repeated measurements

Rating for total score:

High quality = 5-6

Low quality = 3-4

Very low quality = 0-2

Supplementary table S2. Quality assessment of the included meta-analysis/systematic reviews using the Assessment of Multiple Systematic Reviews (AMSTAR) rating tool.

Reviews	1	2	3	4	5	6	7	8	9	10	11	Rating	Quality*
Mayorga-Vega et al. 2014 [1]	Yes	No	Yes	Yes	No	Yes	No	No	Yes	Yes	Yes	7	Medium
Mayorga-Vega et al. 2014 [2]	Yes	No	Yes	Yes	No	Yes	No	No	Yes	Yes	Yes	7	Medium
Bennet et al. 2016 [3]	Yes	Yes	Yes	Yes	No	Yes	Yes	Yes	N/A	N/A	Yes	8	High
Mayorga-Vega et al. 2015 [4]	Yes	Yes	Yes	Yes	No	Yes	No	No	Yes	Yes	Yes	8	High
Mayorga-Vega et al. 2016 [5]	Yes	Yes	Yes	Yes	No	Yes	No	No	Yes	Yes	Yes	8	High

AMSTAR contains 11-items to appraise the methodological aspects of the systematic reviews. All 11-items were scored as “Yes”, “No”, “Can’t Answer” (C/A) or “Not Applicable” (N/A). A total possible score of 11 was calculated, counting only for positive responses ("Yes").

*The final quality rates were computed by tertiles, where the first tertile ranged from 0 to 3 points, the second tertile from 4 to 7 points and the third tertile from 8 to 11 points. Likewise, each tertile were treated as “low”, “medium” or “high”, quality, respectively.

REFERENCES

Mayorga-Vega D, Merino-Marban R, Viciana J Criterion-related validity of sit-and-reach tests for estimating hamstring and lumbar extensibility: a meta-analysis. *J Sports Sci Med* 2014;13:1-14.

Mayorga-Vega D, Viciana J, Cocca A *et al.* Criterion-related validity of toe-touch test for estimating hamstring extensibility: A metaanalysis. *J Hum Sport Exerc* 2014;9:188-200.

Bennett H, Parfitt G, Davison K *et al.* Validity of submaximal step tests to estimate maximal oxygen uptake in healthy adults. *Sports Med* 2016;46:737-750.

Mayorga-Vega D, Aguilar-Soto P, Viciana J Criterion-related validity of the 20-m shuttle run test for estimating cardiorespiratory fitness: a meta-analysis. *J Sports Sci Med* 2015;14:536-547.

Mayorga-Vega D, Bocanegra-Parrilla R, Ornelas M *et al.* Criterion-related validity of the distance- and time-based walk/run field tests for estimating cardiorespiratory fitness: a systematic review and Meta-analysis. *PLoS One* 2016;11:e0151671-e0151671.

Supplementary table S3. Quality assessment of fitness criterion-related validity studies.
NA indicates not applicable because the criterion-related validity of the study is carried out on the dynamometer, not in subjects.

Study	Fitness Component	Number of study subjects	Description of the study population	Statistical analysis	Total score
Leger & Boucher 1980 [6]	Cardiorespiratory fitness	1	0	1	2
Santa María et al. 1976 [7]	Cardiorespiratory fitness	1	2	0	3
Siconolfi et al. 1985 [8]	Cardiorespiratory fitness	1	1	1	3
Ritchie et al. 2005 [9]	Cardiorespiratory fitness	1	2	0	3
Azmi & Sulaiman, 2017 [10]	Cardiorespiratory fitness	1	2	0	3
McGawley 2017 [11]	Cardiorespiratory fitness	0	1	2	3
Giacomantonio et al. 2020 [12]	Cardiorespiratory fitness	1	2	0	3
Leger et al. 1988 [13]	Cardiorespiratory fitness	2	1	1	4
Flouris et al. 2010 [14]	Cardiorespiratory fitness	1	2	1	4
Gabriel et al. 2010 [15]	Cardiorespiratory fitness	2	2	0	4
Hansen et al. 2011 [16]	Cardiorespiratory fitness	2	2	0	4
Burr et al. 2011 [17]	Cardiorespiratory fitness	1	2	1	4
Mikawa & Senjyu et al. 2011 [18]	Cardiorespiratory fitness	2	2	0	4
Arcuri et al. 2015 [19]	Cardiorespiratory fitness	2	2	0	4
Jurgensen et al. 2015 [20]	Cardiorespiratory fitness	2	2	0	4
Lerche et al. 2017 [21]	Cardiorespiratory fitness	2	2	0	4
Siconolfi et al. 1982 [22]	Cardiorespiratory fitness	2	2	1	5
Kline et al. 1987 [23]	Cardiorespiratory fitness	2	2	1	5
Oja et al. 1991 [24]	Cardiorespiratory fitness	2	2	1	5
Laukkanen et al. 1992 [25]	Cardiorespiratory fitness	2	2	1	5
Weller et al. 1992 [26]	Cardiorespiratory fitness	2	2	1	5

Laukkanen et al. 1993 [27]	Cardiorespiratory fitness	2	2	1	5
George et al. 1993 [28]	Cardiorespiratory fitness	2	2	1	5
George et al. 1998 [29]	Cardiorespiratory fitness	2	2	1	5
McNaughton et al. 1998 [30]	Cardiorespiratory fitness	1	2	2	5
Greenhalgh et al. 2001 [31]	Cardiorespiratory fitness	1	2	2	5
Larsen et al. 2002 [32]	Cardiorespiratory fitness	2	2	1	5
Flouris et al. 2004 [33]	Cardiorespiratory fitness	1	2	2	5
Cooper et al. 2005 [34]	Cardiorespiratory fitness	1	2	2	5
Kumar et al. 2012 [35]	Cardiorespiratory fitness	1	2	2	5
Seneli et al. 2013 [36]	Cardiorespiratory fitness	1	2	2	5
Carvalho et al. 2015 [37]	Cardiorespiratory fitness	1	2	1	5
Teren et al. 2016 [38]	Cardiorespiratory fitness	2	2	1	5
Guo et al. 2018 [39]	Cardiorespiratory fitness	1	2	2	5
Manttari et al. 2018 [40]	Cardiorespiratory fitness	2	2	1	5
Ricci et al. 2019 [41]	Cardiorespiratory fitness	1	2	2	5
Bonet et al. 2020 [42]	Cardiorespiratory fitness	1	2	2	5
Kieu et al. 2020 [43]	Cardiorespiratory fitness	1	2	2	5
Dolgener et al. 1994 [44]	Cardiorespiratory fitness	2	2	2	6
Laukkanen et al. 2000 [45]	Cardiorespiratory fitness	2	2	2	6
Flouris et al. 2005 [46]	Cardiorespiratory fitness	2	2	2	6
Metsios et al. 2008 [47]	Cardiorespiratory fitness	2	2	2	6
Kim et al. 2011 [48]	Cardiorespiratory fitness	2	2	2	6
Aadahl et al. 2012 [49]	Cardiorespiratory fitness	2	2	2	6
Cao et al. 2013 [50]	Cardiorespiratory fitness	2	2	2	6

Lunt et al. 2013 [51]	Cardiorespiratory fitness	2	2	2	6
Beutner et al. 2015 [52]	Cardiorespiratory fitness	2	2	2	6
Di Thommazo-Luporini et al. 2015 [53]	Cardiorespiratory fitness	2	2	2	6
Di Thommazo-Luporini et al. 2016 [54]	Cardiorespiratory fitness	2	2	2	6
Hansen et al. 2016 [55]	Cardiorespiratory fitness	2	2	2	6
Jamnick et al. 2016 [56]	Cardiorespiratory fitness	2	2	2	6
Jurio-Iriarte et al. 2017 [57]	Cardiorespiratory fitness	2	2	2	6
Jurio-Iriarte et al. 2018 [58]	Cardiorespiratory fitness	2	2	2	6
Hong et al. 2019 [59]	Cardiorespiratory fitness	2	2	2	6
Lee et al. 2019 [60]	Cardiorespiratory fitness	2	2	2	6
Lima et al. 2019 [61]	Cardiorespiratory fitness	2	2	2	6
Berger 1966 [62]	Musculoskeletal fitness	1	0	0	1
Wells & Dillon 1952 [63]	Musculoskeletal fitness	2	0	0	2
DeWitt 1944 [64]	Musculoskeletal fitness	2	0	0	2
Mathews 1957 [65]	Musculoskeletal fitness	2	0	0	2
Harvey & Scott 1967 [66]	Musculoskeletal fitness	2	0	0	2
Knudson & Johnston 1995 [67]	Musculoskeletal fitness	1	1	0	2
Clemons et al. 2004 [68]	Musculoskeletal fitness	2	0	0	2
Wood & Baumgartner 2004 [69]	Musculoskeletal fitness	2	0	0	2
Clemons et al. 2010 [70]	Musculoskeletal fitness	1	1	0	2
Clemons et al. 2014 [71]	Musculoskeletal fitness	1	0	1	2
Cotten 1972 [72]	Musculoskeletal fitness	2	1	0	3
Jackson & Langford 1989 [73]	Musculoskeletal fitness	2	0	1	3

Kipper & Parker 1987 [74]	Musculoskeletal fitness	1	2	0	3
Liemohn et al. 1994 [75]	Musculoskeletal fitness	1	1	1	3
Diener et al. 1995 [76]	Musculoskeletal fitness	1	2	0	3
Mannion et al. 1997 [77]	Musculoskeletal fitness	1	0	2	3
Mathiowetz 2002 [78]	Musculoskeletal fitness	2	1	0	3
Baumgartner & Gaunt 2005 [79]	Musculoskeletal fitness	2	1	0	3
Ritchie et al. 2005 [9]	Musculoskeletal fitness	1	2	0	3
López-Miñarro et al. 2015 [80]	Musculoskeletal fitness	2	0	1	3
Silva et al. 2015 [81]	Musculoskeletal fitness	1	2	0	3
Broer & Galles 1958 [82]	Musculoskeletal fitness	2	2	0	4
Hall et al. 1992 [83]	Musculoskeletal fitness	2	2	0	4
Harkonen 1993 [84]	Musculoskeletal fitness	NA	NA	1	***Low***
Sparto et al. 1997 [85]	Musculoskeletal fitness	0	2	2	4
Knudson et al. 2001 [86]	Musculoskeletal fitness	1	2	1	4
Youdas et al. 2008 [87]	Musculoskeletal fitness	2	2	0	4
Bohannon et al. 2010 [88]	Musculoskeletal fitness	2	1	1	4
Ayala et al. 2012 [89]	Musculoskeletal fitness	1	2	1	4
Mier et al. 2013 [90]	Musculoskeletal fitness	2	2	0	4
Taylor et al. 2013 [91]	Musculoskeletal fitness	1	2	1	4
Ten Hoor et al. 2016 [92]	Musculoskeletal fitness	1	2	1	4
Clemons et al. 2018 [93]	Musculoskeletal fitness	1	2	1	4

Applegate et al. 2019 [94]	Musculoskeletal fitness	1	2	1	4
Mannion et al. 1994 [95]	Musculoskeletal fitness	2	2	1	5
Kankaanpää et al. 1998 [96]	Musculoskeletal fitness	2	2	1	5
Shechtman et al. 2005 [97]	Musculoskeletal fitness	2	2	1	5
Coorevits et al. 2008 [98]	Musculoskeletal fitness	1	2	2	5
Bui et al. 2015 [99]	Musculoskeletal fitness	1	2	2	5
De Blaiser et al. 2018 [100]	Musculoskeletal fitness	1	2	2	5
Kawano et al. 2010 [101]	Musculoskeletal fitness	2	2	2	6
España-Romero et al. 2010 [102]	Musculoskeletal fitness	NA	NA	2	***High***
Cadenas-Sánchez et al. 2016 [103]	Musculoskeletal fitness	NA	NA	2	***High***
Kolimechkov et al. 2020 [104]	Musculoskeletal fitness	NA	NA	2	***High***
Ritchie et al. 2005[9]	Motor fitness	1	2	0	3
Miyamoto et al. 2008[a] [105]	Motor fitness	2	1	1	4
Miyamoto et al. 2008b [106]	Motor fitness	2	2	0	4

Total score indicates high quality = 5-6; low quality = 3-4; very low quality = 0-2.

REFERENCES

Leger L, Boucher R An indirect continuous running multistage field test: the Universite de Montreal track test. *Can J Appl Sport Sci* 1980;5:77-84.

Santa Maria D, Kinnear GR, Kearney JT *et al.* The objectivity, reliability, and validity of the OSU step test for college males. *Res Q* 1976;47:445-452.

Siconolfi SF, Garber CE, Lasater TM *et al.* A simple, valid step test for estimating maximal oxygen uptake in epidemiologic studies. *Am J Epidemiol* 1985;121:382-390.

Ritchie C, Trost SG, Brown W *et al.* Reliability and validity of physical fitness field tests for adults aged 55 to 70 years. *J Sci Med Sport* 2005;8:61-70.

Azmi SH, Sulaiman N Validity of selected cardiovascular field-based test among Malaysian healthy female adult. *J Fundam Appl Sci* 2017;9:1227-1235.

McGawley K The Reliability and Validity of a Four-Minute Running Time-Trial in Assessing VO2max and Performance. *Front Physiol* 2017;8:270.

Giacomantonio N, Morrison P, Rasmussen R *et al.* Reliability and validity of the 6-minutestep test for clinical assessment of cardiorespiratory fitness in people at risk of cardiovascular disease. *J Strength Cond Res* 2020;34:1376-1382.

Leger LA, Mercier D, Gadoury C *et al.* The multistage 20 metre shuttle run test for aerobic fitness. *J Sports Sci* 1988;6:93-101.

Flouris AD, Metsios GS, Famisis K *et al.* Prediction of VO2max from a new field test based on portable indirect calorimetry. *J Sci Med Spor* 2010;13:70-73.

Gabriel KKP, Rankin RL, Lee C *et al.* Test-retest reliability and validity of the 400-meter walk test in healthy, middle-aged women. *J Phys Act Health* 2010;7:649-657.

Hansen D, Jacobs N, Bex S *et al.* Are fixed-rate step tests medically safe for assessing physical fitness? *Eur J Appl Physiol* 2011;111:2593-2599.

Burr JF, Bredin SS, Faktor MD *et al.* The 6-minute walk test as a predictor of objectively measured aerobic fitness in healthy working-aged adults. *Phys Sportsmed* 2011;39:133-139.

Mikawa K, Senjyu H Development of a field test for evaluating aerobic fitness in middle-aged adults: Validity of a 15-m Incremental Shuttle Walk and Run Test. *J Sports Sci Med* 2011;10:712-717.

Arcuri JF, Borghi-Silva A, Labadessa IG *et al.* Validity and reliability of the 6-minute step test in healthy individuals: a cross-sectional study. *Clin J Sport Med* 2016;26:69-75.

Jurgensen SP, Trimer R, Dourado VZ *et al.* Shuttle walking test in obese women: test-retest reliability and concurrent validity with peak oxygen uptake. *Clin Physiol Funct Imaging* 2015;35:120-126.
Lerche L, Olsen A, Petersen KEN *et al.* Validity of physical activity and cardiorespiratory fitness in the Danish cohort "Diet, Cancer and Health-Next Generations". *Scand J Med Sci Sports* 2017;27:1864-1872.
Siconolfi SF, Cullinane EM, Carleton RA *et al.* Assessing VO2max in epidemiologic studies: modification of the Astrand-Rhyming test. *Med Sci Sports Exerc* 1982;14:335-338.
Kline C, Porcari JP, Hintermeister R *et al.* Estimation of from a one-mile track walk, gender, age and body weight. *Med Sports Exerc* 1987;19:253-259.
Oja P, Laukkanen R, Pasanen M *et al.* A 2-km walking test for assessing the cardiorespiratory fitness of healthy adults. *Int J Sports Med* 1991;12:356-362.
Laukkanen R, Oja P, Pasanen M *et al.* Validity of a two kilometre walking test for estimating maximal aerobic power in overweight adults. *Int J Obes Relat Metab Disord* 1992;16:263-268.
Weller IM, Thomas SG, Cox MH *et al.* A study to validate the Canadian Aerobic Fitness Test. *Can J Public Health* 1992;83:120-124.
Laukkanen RMT, Oja P, Pasanen ME *et al.* Criterion validitv of a two-kilometer walking test for predicting the maximal oxygen uptake of moderately to highly active middle-aged adults. *Scand J Med Sci Sports* 1993: 267-272.
George JD, Vehrs PR, Allsen PE *et al.* Development of a submaximal treadmill jogging test for fit college-aged individuals. *Med Sci Sports Exerc* 1993;25:643-647.
George JD, Fellingham GW, Fisher AG A modified version of the Rockport Fitness Walking Test for college men and women. *Res Q Exerc Sport* 1998;69:205-209.
McNaughton L, Hall P, Cooley D Validation of several methods of estimating maximal oxygen uptake in young men. *Percept Mot Skills* 1998;87:575-584.
Greenhalgh HA, George JD, Hager RL Cross-validation of a quarter-mile walk test using two VO2 max regression models. *Meas Phys Educ Exerc Sci* 2001;5:139-151.
Larsen GE, George JD, Alexander JL *et al.* Prediction of maximum oxygen consumption from walking, jogging, or running. *Res Q Exerc Sport* 2002;73:66-72.
Flouris AD, Koutedakis Y, Nevill A *et al.* Enhancing specificity in proxy-design for the assessment of bioenergetics. *J Sci Med Sport* 2004;7:197-204.
Cooper SM, Baker JS, Tong RJ *et al.* The repeatability and criterion related validity of the 20 m multistage fitness test as a predictor of maximal oxygen uptake in active young men. *Br J Sports Med* 2005;39:e19.

Kumar SK, Khare P, Jaryal AK *et al.* Validity of heart rate based nomogram fors estimation of maximum oxygen uptake in Indian population. *Indian J Physiol Pharmacol* 2012;56:279-283.
Seneli RM, Ebersole KT, O'Connor KM *et al.* Estimated VO2max from the Rockport Walk Test on a Nonmotorized Curved Treadmill. *J Strength Cond Res* 2013;27:3495-3505.
Carvalho LP, Di Thommazo-Luporini L, Aubertin-Leheudre M *et al.* Prediction of cardiorespiratory fitness by the six-minute step test and its association with muscle strength and power in sedentary obese and lean young women: a cross-sectional study. *PLoS One* 2015;10:e0145960.
Teren A, Zachariae S, Beutner F *et al.* Incremental value of veterans specific activity questionnaire and the ymca-step test for the assessment of cardiorespiratory fitness in population-based studies. *Eur J Prev Cardiol* 2016;23:1221-1227.
Guo Y, Bian J, Li Q *et al.* A 3-minute test of cardiorespiratory fitness for use in primary care clinics. *PLoS One* 2018;13:e0201598.
Manttari A, Suni J, Sievanen H *et al.* Six-minute walk test: a tool for predicting maximal aerobic power (VO2 max) in healthy adults. *Clin Physiol Funct Imaging* 2018.
Ricci PA, Cabiddu R, Jürgensen SP *et al.* Validation of the two-minute step test in obese with comorbibities and morbidly obese patients. *Braz J Med Biol Res* 2019;52:e8402.
Bonet JB, Magalhaes J, Viscor G *et al.* A field tool for the aerobic power evaluation of middle-aged female recreational runners. *Women Health* 2020.
Kieu NTV, Jung SJ, Shin SW *et al.* The validity of the YMCA 3-minute step test for estimating maximal oxygen uptake in healthy Korean and Vietnamese adults. *J Lifestyle Med* 2020;10:21-29.
Dolgener FA, Hensley LD, Marsh JJ *et al.* Validation of the Rockport Fitness Walking Test in college males and females. *Res Q Exerc Sport* 1994;65:152-158.
Laukkanen RMT, Kukkonen-Harjula TK, Oja P *et al.* Prediction of change in maximal aerobic power by the 2-km walk test after walking training in middle-aged adults. *Int J Sports Med* 2000;21:113-116.
Flouris AD, Metsios GS, Koutedakis Y Enhancing the efficacy of the 20 m multistage shuttle run test. *Br J Sports Med* 2005;39:166-170.
Metsios GS, Flouris AD, Koutedakis Y *et al.* Criterion-related validity and test-retest reliability of the 20m square shuttle test. *J Sci Med Sport* 2008;11:214-217.
Kim J, Jung SH, Cho HC Validity and Reliability of Shuttle-Run Test in Korean Adults. *Int J Sports Med* 2011;32:580-585.
Aadahl M, Zacho M, Linneberg A *et al.* Comparison of the Danish step test and the watt-max test for estimation of maximal oxygen uptake: the Health2008 study. *Eur J Prev Cardiol*
2013;20:1088-1094.

Cao Z-B, Miyatake N, Aoyama T *et al.* Prediction of maximal oxygen uptake from a 3-minute walk based on gender, age, and body composition. *J Phys Act Health* 2013;10:280-287.
Lunt H, Roiz De Sa D, Roiz De Sa J *et al.* Validation of one-mile walk equations for the estimation of aerobic fitness in British military personnel under the age of 40 years. *Mil Med* 2013;178:753-759.
Beutner F, Ubrich R, Zachariae S *et al.* Validation of a brief step-test protocol for estimation of peak oxygen uptake. *Eur J Prev Cardiol* 2015;22:503-512.
Di Thommazo-Luporini L, Pinheiro Carvalho L, Luporini R *et al.* The six-minute step test as a predictor of cardiorespiratory fitness in obese women. *Eur J Phys Rehabil Med* 2015;51:793-802.
Di Thommazo-Luporini L, Carvalho LP, Luporini RL *et al.* Are cardiovascular and metabolic responses to field walking tests interchangeable and obesity-dependent? *Disabil Rehabil* 2016;38:1820-1829.
Hansen D, Jacobs N, Thijs H *et al.* Validation of a single-stage fixed-rate step test for the prediction of maximal oxygen uptake in healthy adults. *Clin Physiol Funct Imaging* 2016;36:401-406.
Jamnick NA, By S, Pettitt CD *et al.* Comparison of the YMCA and a custom submaximal exercise test for determining Vo2max. *Med Sci Sports Exerc* 2016;48:254-259.
Jurio-Iriarte B, Gorostegi-Anduaga I, Rodrigo Aispuru G *et al.* Association between Modified Shuttle Walk Test and cardiorespiratory fitness in overweight/obese adults with primary hypertension: EXERDIET-HTA study. *J Am Soc Hypertens* 2017;11:186-195.
Jurio-Iriarte B, Brubaker PH, Gorostegi-Anduaga I *et al.* Validity of the modified shuttle walk test to assess cardiorespiratory fitness after exercise intervention in overweight/obese adults with primary hypertension. *Clin Exp Hypertens* 2018:1-6.
Hong SH, Yang HI, Kim DI *et al.* Validation of submaximal step tests and the 6-min walk test for predicting maximal oxygen consumption in young and healthy participants. *Int J Environ Res Public Health* 2019;16.
Lee O, Lee S, Kang M *et al.* Prediction of maximal oxygen consumption using the Young Men's Christian Association-step test in Korean adults. *Eur J Appl Physiol* 2019;119:1245-1252.
Lima LP, Leite HR, Matos MA *et al.* Cardiorespiratory fitness assessment and prediction of peak oxygen consumption by Incremental Shuttle Walking Test in healthy women. *PLoS One* 2019;14:e0211327.
Berger R Evaluation of the 2-minute sit-up test as a measure of muscular endurance and strength. *J Assoc Phys Ment Rehabil* 1966;20:140.
Wells KF, Dillon EK The sit-and-reach. A test of back and leg flexibility. *Res Q Exerc Sport* 1952;23:115-118.
DeWitt R A study of the sit-up Type of test as a means of measuring strength and endurance of the abdominal muscles. *Res Q* 1944;15:60-63.
Mathews DK, Shaw V, Bohnen M Hip flexibility of college women as related to length of body segments. *Res Q* 1957;28:352-356.
Harvey VP, Scott GD An investigation of the curl-down test as a measure of abdominal strength. *Res Q* 1967;38:22-27.
Knudson D, Johnston D Validity and reliability of a bench trunk-curl test of abdominal endurance. *J Strength Cond Res* 1995;9:165-169.

Clemons JM, Duncan CA, Blanchard OE *et al.* Relationships between the flexed-arm hang and select measures of muscular fitness. *J Strength Cond Res* 2004;18:630-636.
Wood HM, Baumgartner TA Objectivity, reliability, and validity of the bent-knee push-up for college-age women. *Meas Phys Educ Exerc Sci* 2004;8:203-212.
Clemons JM, Campbell B, Jeansonne C Validity and reliability of a new test of upper body power. *J Strength Cond Res* 2010;24:1559-1565.

Clemons JM Construct validity of a modification of the flexed arm hang test. *J Strength Cond Res* 2014;28:3523-3530.
Cotten DJ A comparison of selected trunk flexibility tests. *Am Correct Ther J* 1972;26:24-26.
Jackson A, Langford NJ The criterion-related validity of the sit and reach test: replication and extension of previous findings. *Res Q Exerc Sport* 1989;60:384-387.
Kippers V, Parker AW Toe-touch test: A measure of its validity. *Phys Ther* 1987;67:1680-1684.
Liemohn W, Sharpe GL, Wasserman JF Criterion related validity of the sit-and-reach test. *J Strength Cond Res* 1994;8:91-94.
Diener MH, Golding LA, Diener D Validity and reliability of a one-minute half sit-up test of abdominal strength and endurance. *Res Sports Med* 1995;6:105-119.
Mannion AF, Connolly B, Wood K *et al.* The use of surface EMG power spectral analysis in the evaluation of back muscle function. *J Rehabil Res Dev* 1997;34:427-439.
Mathiowetz V Comparison of Rolyan and Jamar dynamometers for measuring grip strength. *Occup Ther Int* 2002;9:201-209.
Baumgartner TA, Gaunt SJ Construct related validity for the Baumgartner Modified pull-up test. *Meas Phys Educ Exerc Sci* 2005;9:51-60.
López-Miñarro P, Vaquero-Cristóbal R, Muyor JM *et al.* Criterion-realted validity of sit-and-reach test as measure of hamstring extensibility in older women. *Nutr Hosp* 2015;32:312-317.
Silva P, Franco J, Gusmao A *et al.* Trunk strength is associated with sit-to-stand performance in both stroke and healthy subjects. *Eur J Phys Rehabil Med* 2015;51:717-724.
Broer MR, Galles NRG Importance of relationship between various body measurements in performance of the toe-touch test. *Res Q* 1958;29:253-263.
Hall GL, Hetzler RK, Perrin D *et al.* Relationship of timed sit-up tests to isokinetic abdominal strength. *Res Q Exerc Sport* 1992;63:80-84.
Härkönen R, Harju R, Alaranta H Accuracy of the Jamar dynamometer. *J Hand Ther* 1993;6:259-262.
Sparto PJ, Parnianpour M, Reinsel TE *et al.* Spectral and temporal responses of trunk extensor electromyography to an isometric endurance test. *Spine (Phila Pa 1976)* 1997;22:418-425; discussion 425-426.
Knudson D The validity of recent curl-up tests in young adults. *J Strength Cond Res* 2001;15:81-85.

Youdas JW, Krause DA, Hollman JH Validity of hamstring muscle length assessment during the sit-and-reach test using an inclinometer to measure hip joint angle. *J Strength Cond Res* 2008;22:303-309.
Bohannon RW, Bubela DJ, Magasi SR *et al.* Sit-to-stand test: Performance and determinants across the age-span. *Isokinet Exerc Sci* 2010;18:235-240.
Ayala F, Sainz de Baranda P, De Ste Croix M *et al.* Reproducibility and concurrent validity of hip joint angle test for estimating hamstring flexibility in recreationally active young me. *J Strength Cond Res* 2012;26:2372-2382.
Mier CM, Shapiro BS Sex differences in pelvic and hip flexibility in men and women matched for sit-and-reach score. *J Strength Cond Res* 2013;27:1031-1035.
Taylor JD, Fletcher JP Correlation between the 8-repetition maximum test and isokinetic dynamometry in the measurement of muscle strength of the knee extensors: A concurrent validity study. *Physiother Theory Pract* 2013;29:335-341.
Ten Hoor GA, Musch K, Meijer K *et al.* Test-retest reproducibility and validity of the back-leg-chest strength measurements. *Isokinet Exerc Sci* 2016;24:209-216.
Clemons J Construct Validity of Two Different Methods of Scoring and Performing Push-ups. *J Strength Cond Res* 2019;33:2971-2980.
Applegate ME, France CR, Russ DW *et al.* Sorensen test performance is driven by different physiological and psychological variables in participants with and without recurrent low back pain. *J Electromyogr Kinesiol* 2019;44:1-7.
Mannion AF, Dolan P Electromyographic median frequency changes during isometric contraction of the back extensors to fatigue. *Spine (Phila Pa 1976)* 1994;19:1223-1229.
Kankaanpää M, Laaksonen D, Taimela S *et al.* Age, sex, and body mass index as determinants of back and hip extensor fatigue in the isometric Sørensen back endurance test. *Arch Phys Med Rehabil* 1998;79:1069-1075.
Shechtman O, Gestewitz L, Kimble C Reliability and validity of the DynEx dynamometer. *J Hand Ther* 2005;18:339-347.
Coorevits P, Danneels L, Cambier D *et al.* Assessment of the validity of the Biering-Sørensen test for measuring back muscle fatigue based on EMG median frequency characteristics of back and hip muscles. *J Electromyogr Kinesiol* 2008;18:997-1005.
Bui HT, Farinas M-I, Fortin A-M *et al.* Comparison and analysis of three different methods to evaluate vertical jump height. *Clin Physiol Funct Imaging* 2015;35:203-209.
De Blaiser C, De Ridder R, Willems T *et al.* Reliability and validity of trunk flexor and trunk extensor strength measurements using handheld dynamometry in a healthy athletic population. *Phys Ther Sport* 2018;34:180-186.
Kawano MM, Ambar G, Oliveira BIR *et al.* Influence of the gastrocnemius muscle on the sit-and-reach test assessed by angular kinematic analysis. *Braz J Phys Ther* 2010;14:10-15.
España-Romero V, Ortega FB, Vicente-Rodríguez G *et al.* Elbow position affects handgrip strength in adolescents: validity and reliability of Jamar, DynEx, and TKK dynamometers. *J Strength Cond Res* 2010;24:272-277.

Cadenas-Sanchez C, Sanchez-Delgado G, Martinez-Tellez B *et al.* Reliability and validity of different models of TKK hand dynamometers. *Am J Occup Ther* 2016;70:7004300010.
Kolimechkov S, Castro-Piñero J, Petrov A *et al.* The effect of elbow position on the handgrip strength test in children: validity and reliability of TKK 5101 and DynX dynamometers. *Pedagogy Phys Cult Sports* 2020;24:240-247.
Miyamoto K, Takebayashi H, Takimoto K *et al.* The criterion-related validity of the ten step test compared with motor reaction time. *J Phys Ther Sci* 2008;20:261-265.
Miyamoto K, Takebayashi H, Takimoto K *et al.* A new simple performance test focused on agility in elderly people: The Ten Step Test. *Gerontology* 2008;54:365-372.

Supplementary table S4. Overview of field-based fitness tests criterion-related validity studies in adults.

Author	Participants	Age	Field-based Test	Gold standard	Statistical methods	Main outcome	Conclusion
Very low quality studies							
Cardiorespiratory fitness							
Leger & Boucher 1980 [6]	Adults=25 (Healthy)	24.4±2.8	The University Montreal track (UM-track)	Gas analyzer in maximal treadmill test (Modified Balke protocol)	Correlation coefficient (r), SEE	r=0.96 ($p<0.05$), SEE= 2.81 ml/kg/min, between UM-track test and VO_{2max}.	The UM-track test is accurate, valid and safe for young and middle-age adults, males and females, whether they are trained or not.
Musculoskeletal fitness (isometric)							
Clemons et al. 2014 [71]	Females =31 (Healthy)	19-36	Modified flexed-arm hang	1RM (Absolute and relative isometric strength)	Correlation coefficient (r), linear regression	r =0.10 (p=0.61) between modified flexed arm hang and absolute isometric strength. r=0.88 (p< 0.001) between modified flexed arm hang and relative isometric strength.	Modified flexed-arm hang scores were not related to absolute strength, however, is a valid estimate of the construct of relative isometric strength.
Musculoskeletal fitness (endurance)							
Clemons et al. 2004 [68]	Females=60 (Healthy)	20.7±2.15	Flexed-arm hang	1RM (absolute strength) 1RM/mass (relative strength)	Correlation coefficient (r)	r ranges from -0.14 to 0.72 between flexed-arm hang and the different criterion measures.	No significant relationship was found between any of the flexed-arm hang variations and absolute strength or muscle

				Repetitions at 70% of 1RM (muscle endurance)		r =0.72 ($p<0.01$) between U90º variation and relative strength.	endurance; however, all flexed-arm hang variations correlated significantly with relative strength.
Wood & Baumgartner 2004 [69]	Females= 87<br>(Healthy)	College	Bent-knee push-up<br>Revised push-up	1RM bench press	Correlation coefficient (r)	r=0.67 between bent-knee push-up and bench press.<br>r=0.68 between revised push-up and bench press.	The validity for the bent-knee push-up test is acceptable, however, somewhat more questionable. Because the validity coefficient for both the bent-knee and the revised push-up tests are similar, either test can be used.
Harvey & Scott 1967 [66]	Females =60<br>(Healthy)	College	Modified curl-up (30 s)<br>Curl-up	Dynamometer (Elgin Multiple Angle)	Correlation coefficient (r)	r=0.44 ($p<0.05$) between modified curl-up and dynamometer scores.<br>r=0.32 ($p<0.05$) between curl-up and dynamometer scores.	Both tests have low validity.
Berger 1966 [62]	Adults= 47<br>(Healthy)	College	Two-minute sit-up	1 RM sit-up	Correlation coefficient (r)	r=0.51 ($p<0.05$) between two-minute sit-up and 1-RM sit-up.	The two-minute sit-up test is not valid to assess endurance of the abdominal muscles.
Knudson & Johnston 1995 [67]	Females = 10<br>Males= 10<br>(Healthy)	19-32	Modified curl-up (2 min, legs elevated on a bench, hands interlocke	Cibex isokinetic dynamometer	Correlation coefficient (r)	r= 0.38 ($p=0.07$) between the modified curl-up and isokinetic dynamometer.	Modified curl-up test has low validity.

			d on the abdomen)				
DeWitt 1944 [64]	Males = 102 (Healthy)	College	1º Modified curl-up (touch knees with opposites elbow)<br>2º Modified curl-up (from setting, hands interlocked behind the neck, touch knees with opposites elbow)<br>3º Modified curl-up (held feet)	Dynamometer	Correlation coefficient (r)	1º modified curl-up: strength, r=0.04; endurance, r=0.25 with dynamometer.<br>2º modified curl-up: strength, r=0.16; endurance, r=0.37 with dynamometer.<br>3º modified curl-up: strength, r=0.14; endurance, r=0.26 with dynamometer.	Modified curl-up variations have low validity.
Musculoskeletal fitness (explosive)							
Clemons et al. 2010 [70]	Females =24<br>Males=19<br>(Healthy)	22.1±1.82<br>23.1±2.95	Medicine ball put	Bench press power	Correlation coefficient (r)	r=0.86 and 0.79 (p<0.01 for both) for female and male, respectively, between bench press power and medicine ball put.	The medicine ball put test is a valid method to use in assessing upper body power for both college-age males and females.

Musculoskeletal fitness (flexibility)							
Wells & Dillon 1952 [63]	Adults=100<br>(Healthy)	College	Sit-and-reach	The standing bobbing test	Correlation coefficient (r)	r=0.90 between the standing bobbing and sit-and-reach tests.	The sit-and-reach is a valid test of back and leg flexibility.
Mathews 1957<br>[65]	Females =66<br>(Healthy)	College	Adapted Kraus-Weber floor touch test and Wells sit-and-reach	Leighton flexometer	Correlation coefficient (r)	r=0.82 between adapted Kraua-Weber floor touch and Leighton flexometer.<br>r=0.74 between Wells sit-and-reach and Leighton flexometer.	Adapted Kraua-Weber floor touch and Wells sit-and-reach tests are valid test to assess hip flexibility.
Low quality studies							
Cardiorespiratory fitness							
Ritchie et al. 2005 [9]	Females = 10<br>Males = 13<br>(Healthy)	55-70	2-level step	Gas analyzer in submaximal cycle test	Correlation coefficient (r)	r= -0.60 ($p<0.001$) between step test and submaximal exercise test.	The 2-level step test was found to be a valid test.
Giacomantonio et al. 2020 [12]	Females = 16<br>Males = 12<br>(Risk of cardiovascular disease)	>17	6-minute step	Gas analyzer in maximal treadmill test	Correlation coefficient (r), CI	r=0.88 (CI: 0.57-0.97) between 6-minute step test and VO_{2peak}.	The 6-minute step test seems to be a valid option for assessing cardiorespiratory fitness of people at risk of cardiovascular disease.
Arcuri et al. 2015 [19]	Females=49<br>Males=42<br>(Healthy)	18-86	6-minute single 20-cm step	6-minute walk test	Correlation coefficient (r)	r=0.72 ($p<0.001$)	Six-minute single 20-cm step test is a valid test.

Siconolfi et al. 1985 [8]	Females= 19<br>Males= 29<br>(Healthy)	19-70	3-minute single 25.4-cm step	Gas analyzer in submaximal cycle test (Modified Astrand-Rhyming protocol)	Correlation coefficient (r), SEE	r=0.92 (p=0.01) between step test and directly measured $VO_{2\ max}$.<br>SEE=0.30 ml/kg/min.	The 3-minute single 25.4-cm step test protocol provides valid estimates of cardiorespiratory fitness over a wide age range.
Santa María et al. 1976 [7]	Males= 21<br>(Healthy)	22.54±4.12	USO step	Gas analyzer in submaximal cycle test (Astrand method)	Correlation coefficient (r)	r=0.47 between USO step test and $VO_{2max.}$	USO step test seems to have a poor validity.
Hansen et al. 2011 [16]	Females = 60<br>Males = 53<br>(Healthy)	23-75	Modified Harvard step	Gas analyzer in maximal cycle test	Paired t-tests	r =0.54 (p< 0.01) between modified Harvard step test and $VO_{2max.}$	Standardized fixed-rate modified Harvard step test elicited vigorous exercise intensities, especially in small, obese, and/or physically deconditioned subjects.
Lerche et al. 2017 [21]	Females = 65<br>Males=60<br>(Healthy)	20-63	Danish step	Gas analyzer in maximal treadmill test	Correlation coefficient (r)	r=0.56 and 0.66 (p<.001 for both) between Danish step test and $VO_{2max}$for men and women, respectively.	The Danish step test is a valid assessment method for women, however not for men (under estimation of cardiorespiratory fitness).
Leger et al. 1988 [13]	Females = 24<br>Males = 53<br>(Healthy)	18-50	20-m shuttle run	Douglas bag methods	Multiple regression, SEE	r=0.90 between VO_{2max} and maximal speed.<br>Y=31.025+3.238*speed +3.248*age+ 0.1536*speed*age (r=0.71; SEE=5.1 ml/kg/min)	20-m shuttle run test is a valid method to measure $VO_{2\ max}$ in adult population.
Jurgensen et al. 2015 [20]	Females = 46	18-46	Incrementa l shuttle	Gas analyzer in maximal treadmill	Correlation coefficient (r)	r=0.64 (p<0.05).	The incremental shuttle walking test had acceptable

	(Obese)		walking	test			validity is this population.
Mikawa & Senjyu et al. 2011 [18]	Males = 68 (Healthy)	40-59	15-m incrementa l shuttle walk and run<br>1500-m fast walk	Gas analyzer in maximal cycle test	Linear regression	r=0.86 (p<0.01) between 15-m incremental shuttle walk and run test and VO_{2max}.<br>r=0.52 (p<0.01) between 1500-m fast walk test and VO_{2max}.	The 15-m incremental shuttle walk and run test is highly recommended as a field test for evaluating cardiorespiratory fitness in middle-aged adults. The 1500-m fast walk test is not valid test in this population.
Flouris et al. 2010 [14]	Males = 45 (Healthy)	18-29	15-m square shuttle run	Gas analyzer in maximal treadmill test	Correlation coefficients (r), linear regression	r=0.87-0.92 (p<0.001) between 15-msquare shuttle run test and VO_{2max}.	15-m square shuttle run test is a highly valid predictive test for VO_{2max}.
McGawley 2017 [11]	Females = 5 Males = 5 (Healthy)	32±7	4-minute running time trial	Gas analyzer in STEP treadmill test + VER phase	Bland-Altmann method, Two-way repeated measures ANOVA	Significant difference between STEP treadmill test and 4-minute running time trial (p=0.008).<br>Mean difference=1.6±3.6 ml/kg/min (p=0.459) for STEP treadmill test vs 4-minute running time trial.	VO_{2max} values attained during 4-minute running time trial were significantly lower than those attained during STEP.
Azmi & Sulaiman, 2017 [10]	Females =15 (Healthy)	20-24	Harvard step<br>20-m shuttle run<br>2.4-km<br>1-mile walk	Gas analyzer in maximal cycle test (Bruce protocol test)	Correlation coefficient (r)	r=0.31 (p=0.27), between Harvard Step test and VO_{2max}.<br>r=0.64 (p<0.05), between 20-m shuttle run test and VO_{2max}.<br>r=0.42 (p=0.13), between 2.4-km run test and VO_{2max}.<br>r=0.18 (p=0.52), between 1-mile walk test and VO_{2max}.	The most suitable cardiorespiratory test to measure VO_{2max} in healthy females adults is the 20-m shuttle run test.

Gabriel et al. 2010 [15]	Females = 66<br>(Healthy)	45-65	400-m walk	Gas analyzer in maximal treadmill test	Spearman correlation coefficient (ρ)	ρ= -0.43 ($p<0.001$), between 400-m walk test and estimated VO_{2max}.<br>ρ= -0.56 ($p<0.001$), between 400m walk test and measured VO_{2max}.	The 400-m walk test is valid for estimating cardiorespiratory fitness in healthy middle-aged women.
Burr et al. 2011 [17]	Females = 21<br>Males = 23<br>(Healthy)	25–59	6-minute walk	Gas analyzer in maximal treadmill test (Modified Bruce protocol test)<br>Douglas bag methods	Spearman correlation coefficient (r), multiple linear regression	r=0.49 ($p<0.001$), between distance walked and VO_{2max}.<br>R^2=0.72 ($p<0.05$), between prediction equation and VO_{2max}.	The 6-minute walk test is valid for estimating cardiorespiratory fitness.
Musculoskeletal fitness (isometric)							
Mathiowetz 2002 [78]	Females = 30<br>Males = 30<br>(Healthy)	20-50	Rolyan dynamome ter<br>Jamar dynamome ter	Known weights	Correlation coefficient (r), paired t-test	r =0.99 ($p<0.05$), between Rolyan and Jamar dynamometers with known weights.<br>Differences between Rolyan and Jamar dynamometer <2.3% ($p>0.05$).	Rolyan and Jamar dynamometer measure equivalently for practical purposes and are valid test to asses isometric muscular strength.
Harkonen 1993 [84]	NA	NA	Jamar dynamome ter	Known weights	Errors	Error>2 kg was found for dynamometers that were purchased in the years 1985, 1986 and 1987.	Jamar dynamometers seem to be rather accurate.
Musculoskeletal fitness (endurance)							

Baumgartner & Gaunt 2005 [79]	Females = 40<br>Males = 42<br>(Healthy)	21.1±3.8<br>21.0±1.6	Baumgartner modified pull-up	1 RM bench press<br>Modified lateral pull-down	Correlation coefficient (r)	r=0.61females and r=0.67 males, between Baumgartner modified pull-up and bench press.<br>r=0.60 females and r=0.85 males, between Baumgartner modified pull-up and modified lateral pull-down.	Baumgartner modified pull-up test is valid to assess arm and shoulder girdle muscular strength and endurance in college men and women.
Clemons et al. 2018 [93]	Males = 31<br>(Healthy)	18-29	Standard push-up<br>Hand-release push-up	1 RM bench press	Correlation coefficient (r), multiple linear regression	r=0.25 (p=0.18) and r=0.7 (p<0.001) between standard push-up with absolute and relative 1RM.<br>rho= -0.05 (p=0.787) and rho=0.39 (p<0.05) between hand-release push-up with absolute and relative 1RM.	Standard push-up has moderate validity to asses relative upper body endurance strength but not absolute.<br>Hand-release push-up is not valid test to assess absolute and relative upper body endurance strength.
Bohannon et al. 2010 [88]	Females =111<br>Males=70<br>(Healthy)	14-85	5-repetition sit-to-stand	Isokinetic dynamometer strength	Correlation coefficient (r), multiple linear regression	r = -0.54 and -0.56 (p<0.001 for both) between isokinetic strength and 5-repetition sit-to-stand test for 14-85 years and 50-85 years respectively.<br>R^2=0.30 isokinetic strength predicting 5-repetition sit-to-stand test in 14-85 and 50-85 group.	There is a significant relationship between knee extension strength and 5-repetition sit-to-stand test. Nevertheless, knee extension strength alone provides an insufficient explanation of 5-repetition sit-to-stand test performance. Age, body weight, and stature also influence 5-repetition sit-to-stand performance.
Silva et al. 2015 [81]	Stroke = 18	>= 20	5-repetition	Isokinetic dynamometer	Correlation coefficient	r range from -0.41 to -0.51 (all p<0.05) between total	5-repetition sit-to-stand test obtained from low to

	Healthy = 18		sit-to-stand	strength	(r)	duration of 5-repetition sit-to-stand test and strength of the trunk muscles	moderate correlation with trunk muscular strength.
Taylor et al. 2013 [91]	Females = 15<br>Males = 15<br>(Healthy)	23±1	8-repetition maximum test of knee extensors	Isokinetic dynamometer strength	Correlation coefficient (r), linear regression	R^2 = 0.73, 0.73 and 0.51 (all p<0.001) between for peak torques at 60, 120 and 240 degrees per second, respectively, between 8-repetition and isokinetic strength.	8-repetititon maximum testing of knee extensors can be used as a valid measure of lower body muscular strength.
Ten Hoor et al. 2016 [92]	Females = 22<br>Males = 23<br>(Healthy)	18-35	Back-leg-chest	Isokinetic dynamometer strength	Correlation coefficient (r), stepwise linear regression	r range from 0.56 to 0.81 (all p<0.01) and from 0.60 to 0.71 (all p<0.01) between back-leg-chest and criterion measures in males and females respectively.<br>R^2=0.74 for dominant knee extensor and flexor strength predicting back-leg-chest strength.<br>R^2=0.86 for predicting back-leg-chest strength.	Back-leg-chest test can be used to evaluate changes in muscular strength in adults.
Diener et al. 1995 [76]	Females = 21<br>Males = 15<br>(Healthy)	17-68	Modified curl-up (reach 8.89cm with fingertip)	Isometric spine flexion strength (strength table)	Correlation coefficient (r)	r=0.44 (p<0.01) between the modified curl-up test and isometric abdominal strength.	Modified curl-up test is not valid to measure abdominal muscular strength and endurance.

Ritchie et al. 2005 [9]	Females = 10 Males = 13<br>(Healthy)	55-70	Sit-to-stand<br>Lift and reach	1RM bench press,<br>1RM leg press,	Correlation coefficient (r)	r=0.68 (p<0.05) between sit-to-stand test and 1RM leg press.<br>r=0.43 (p>0.05) between lift and reach and 1RM bench press.	Only sit-to-stand was found to be valid when compared 1RM test.<br>Single timed chair raise is not a valid test.
Knudson et al. 2001 [86]	Females = 22<br>Males = 22<br>(Healthy-active)	25.8±5.0	Curl-up<br>Modified curl-up (90s, legs elevated on a bench, hands interlocked on the abdomen)	Isokinetic dynamometer strength	Correlation coefficient (r), linear regression, SEE	r=0.36, SEE=0.10 and r=0.21, SEE=0.11 (p>0.05 for both) between the curl-up and isokinetic muscular strength for men and women, respectively.<br>r=0.50, SEE=0.08 and r=0.46, SEE=0.08 (p<0.05 for both) between the curl-up and isokinetic muscular endurance for men and women, respectively.<br>r=0.07, SEE=0.10 and r= -0.19, SEE=0.10 (p>0.05 for both) between the modified curl-up and isokinetic muscular strength for men and women, respectively.<br>r=0.23, SEE=0.09 and r=0.10, SEE=0.09 (p>0.05 for both) between the modified curl-up and isokinetic muscular endurance for men and women, respectively.	The curl-up test and the modified curl-up have weak validity to assess abdominal muscular strength and endurance.

Hall et al. 1992 [83]	Females = 28<br>Males = 23<br>(Healthy)	22.2±4.6<br>23.1±7.4	1º Modified curl-up (knees extend, hand interlocked behind the neck, held feet, flex up to 90º)<br>2º Modified curl-up (arm folded across the chest, feet held, flex up to 90º)<br>Curl-up	Isokinetic dynamometer strength	Correlation coefficient (r)	1º Modified Curl-up: r=0.42 and 0.40 (p<0.01, for both) in males, for concentric and eccentric peak torque, respectively; and r=-0.18 and -0.21 (p>0.05 for both) in females for concentric and eccentric peak torque, respectively.<br>2º Modified Curl-up: r=-0.07 and -0.08 (p>0.05 for both) in males, for concentric and eccentric peak torque, respectively; and r=-0.41 and -0.38 (p<0.01, for both) in females for concentric and eccentric peak torque, respectively.<br>Curl-up: r=0.27 (p>0.05) and 0.32 (p<0.01) in males, for concentric and eccentric peak torque, respectively; and r=-0.25 and -0.28 (p>0.05 for both) in females for concentric and eccentric peak torque, respectively.	The use of timed curl-up test is not a valid method of estimating isokinetic abdominal muscular strength.
Mannion et al. 1997 [77]	Female = 17<br>Male = 17<br>(healthy)	19-48	Modified Biering-Sørensen	Electromyography	Simple and multiple linear regression, ANOVA/ANCOVA	R^2=0.85 and 0.57, (p<0.05 for both) between time endurance and median frequency of left and right lumbar region.	The Biering-Sørensen test is valid for measuring back muscle fatigue.

						No difference between the greatest power spectrum and endurance time (p>0.05).	
Sparto et al. 1997 [85]	Male = 10<br>(healthy)	20-32	Biering-Sørensen	Electromyography	Linear regression, MANOVA for repeated measurements	R^2=0.88 (p<0.05) between median frequency and time test.<br>There was not significant level among lumbar level, muscle location and endurance time (p>0.05).	The Biering-Sørensen test is valid for measuring back muscle fatigue.
Applegate et al. 2019 [94]	Female = 24<br>Male = 24<br>(Healthy and low back pain (50%))	29.2±2.2<br>(Healthy)<br>24.3±1.5<br>(Low back pain)	Biering-Sørensen	Electromyography	Hierarchical and stepwise linear regression	R^2=0.56 (p<0.001) between time test with normalized median power frequency slope of the erector spinae (β=0.350, p<0.01), the biceps femoris (β=0.375, p<0.01), and self-efficacy (β= 0.437, p<0.01).	The Biering-Sørensen test has moderate validity for measuring back muscle fatigue in healthy adults. In adults with low back pain self-efficacy and trunk mass appears to be the best predictor.
Musculoskeletal fitness (flexibility)							
Jackson & Langford 1989 [73]	Females = 52<br>Males = 52<br>(Healthy)	20-45	Sit-and-reach	Manual goniometer	Correlation coefficients (r)	r=0.70 between the sit-and-reach test and hamstring flexibility; and r=0.12 with low back flexibility, in female respectively.<br>r=0.89 between the sit-and-reach test and hamstring flexibility; and r=0.59 with low back flexibility, in male respectively.	The sit-and-reach test appears to possess moderate validity as a measure of hamstring flexibility, but is not a valid measure of low back flexibility.

Youdas et al. 2008 [87]	Males=106<br>Females=106<br>(Healthy)	20-79	Sit-and-reach	Passive straight leg raise	Correlation coefficient (r)	r = 0.59 (p<0.01) between sit-and-reach test and passive straight leg raise.	Sit-and-reach test is not a valid method to measure hip joint angle.
Mier et al. 2013 [90]	Females = 35<br>Males = 35<br>(Healthy)	23±4	Sit-and-reach	Pelvic angle during sit-and-reach and hip joint angle during passive straight leg raise.	Correlation coefficient (r), paired t-test	r=0.82 (CI: 0.70-0.89) and 0.83 (CI: 0.72-0.90) between sit-and-reach scores and pelvic angle for men and women, respectively.<br>r=0.60 (CI: 0.39-0.75) and 0.56 (CI: 0.33-0.73) between sit-and-reach scores and passive straight leg raise hip joint angle for men and women, respectively.	Sit-and-reach score does not adequately assess spine and pelvic flexibility.
López-Miñarro et al. 2015 [80]	Females=120<br>(Healthy)	62,34±8,75	Sit-and-reach	Passive straight leg raise test	Correlation coefficient (r), linear regression	R^2 =0.50, 0.45 and 0.49 (all p<0.001) between sit-and-reach and straight leg raise test for left, right and both legs, respectively.	The validity of sit-and-reach test as measure of hamstring muscle extensibility on older women is moderate.
Liemohn et al 1994 [75]	Females = 20<br>Males = 20<br>(Healthy)	25.1±6.2<br>24.0±4.6	Sit-and-reach,<br>Modified sit-and-reach (Cailliet)	Lumbar spine range with Inclinometer	Correlation coefficient (r)	r=0.56 and 0.68 (p<0.01, for both) as measures of low back flexibility in females for sit-and-reach and modified tests, respectively.<br>r=0.31 and 0.33 (p>0.05, for both) as measures of low back flexibility in males for sit-and-reach and modified tests, respectively.	The sit-and-reach test and its modified, do not have criterion-related validity as a field test of low back flexion range of motion.

Cotten 1972 [72]	Females= 38<br>Males= 37<br>(Healthy)	College	Sit-and-reach<br>Modified sit-and-reach<br>Cureton test<br>Scott-French bobbing test	Leighton flexometer	Correlation coefficient (r)	r= 0.65 between sit-and-reach and flexometer.<br>r= 0.66 between Modified sit-and-reach and flexometer.<br>r= 0.43 between Cureton test and flexometer.<br>r= 0.52 between Scott-French bobbing test and flexometer.	None of these tests should be considered interchangeable with the Leighton flexometer.
Broer & Galles 1958 [82]	Females=100<br>(Healthy)	18-31	Toe-touch	Leighton Flexometer	Correlation coefficient (r)	r=0.81 between Toe-touch test and flexometer measures.	A study of a group which is known to include more extreme body types is needed for a more detailed validation of the toe-touch test.
Kipper & Parker 1987 [74]	Females =16<br>Males = 17<br>(Healthy)	22.1±3.8<br>21.1±3.0	Toe-touch	Trunk, hip and vertical flexion, were assessed with a camera	Correlation coefficient (r)	r=0.85 ($p<0.001$) between vertical fingertip-floor distances and trunk flexion.<br>r=0.79 ($p < 0.001$) between vertical fingertip-floor distances and hip flexion.<br>r=0.10 between vertical fingertip-floor distances and vertebral flexion.	The vertical fingertip-floor distances a valid measure of hip flexion with the knees extended, although it should not be considered an indication of hip flexion also if the knees are allowed to flex, relieving hamstring muscle tension.
Ayala et al. 2012 [89]	Males=70<br>(Active recreationally)	21.3±2.5	Horizontal hip joint angle	Straight leg raise test with an inclinometer	Multiple linear regression, SEE	Between horizontal hip joint angle test and passive straight leg raise test β = 0.78, SEE=0.10, R^2=0.62, $p<0.01$.	The validity of horizontal hip joint angle test and vertical hip joint angle test is moderate. Furthermore, the horizontal hip joint

			Vertical hip joint angle			Between vertical hip joint angle test and passive straight leg raise test β = 0.80, SEE=0.10, R^2=0.63, p<0.01.	angle test cutoff scores should not be used for vertical hip joint angle test for the detection of short hamstring muscles in young adults.
Motor fitness (balance)							
Miyamoto et al. 2008a [105]	Females = 103<br>Males = 49<br>(Healthy)	71±13	Ten step	Motor reaction time<br>Knee muscle strength<br>Functional reach test	Correlation coefficient (r)	r=0.59 (p<0.01) between ten step test and motor reaction time.<br>r=-0.35 (p<0.01) between ten step test and knee muscle strength.<br>r=-0.42 (p<0.01) between ten step test and functional reach test.	Ten step test is a more useful measurement of agility than previous test batteries.
Miyamoto et al. 2008b [106]	Females = 562<br>Males = 266<br>(Healthy)	22-99	Ten step	Motor reaction time,<br>Single leg standing time	Correlation coefficient (r)	r=0.68 and 0.59 (p<0.01 for both) between ten step test and time supine-to-stand and between ten step test and motor reaction time respectively.	Ten step test is a valid tool to asses agility.
Ritchie et al. 2005 [9]	Females = 10<br>Males = 13	55-70	Romberg test	Change centre of pressure	Correlation coefficient (r)	r ranges from -0.18 to 0.23 (p>0.05 for all) between balance test and change to centre of pressure.	Romberg test were found to be a not valid test to assess static balance.
High quality studies							

Cardiorespiratory fitness

Weller et al. 1992 [26]	Females = 78<br>Males = 76<br>(Healthy)	15-69	Modified Canadian aerobic fitness	Gas analyzer in maximal treadmill test	Linear regression, ICC	R^2=0.75 ($p<0.05$) and ICC=0.81, between prediction equation and $VO2_{max}$.	Modified Canadian aerobic fitness test offers an improved method for estimating VO_{2max}.
Carvalho et al. 2015 [37]	Females=31<br>(Sedentary obese and lean)	20–45	6-minute single 15-cm step	Gas analyzer in submaximal treadmill test	Correlation coefficients (r), multiple linear regression, SEE	r=0.80, 0,79 and 0.31 (all $p<0.05$) between number of step cycles and VO_{2peak} in total sample, lean and obese females.<br>The final model explained 83% of the total variance in VO2peak during treadmill test. The reference equation obtained was: VO_{2peak}= 35.335 - (0.328 x BMI) + (0.069 x NSC)–(0.298 x age), with a SEE=2.9 ml/kg/min (Durbin-Watson test = 2.2; $p<0.01$).	The step cycles of Six-minute single 15-cm step test with BMI, and age, accurately predict VO_{2peak} in both, obese and lean sedentary females.
Teren et al. 2016 [38]	Females = 38<br>Males = 48<br>(Healthy)	45-66	YMCA step	Gas analyzer in maximal treadmill test	Correlation coefficient (r), linear regression	r =0.64 ($p<0.001$) between VO_2 YMCA step test and VO_{2peak}.<br>Age, gender, BMI and YMCA step test explained the 91% of the variance of $VO2_{peak}$ ($p<0.05$).	YMCA step test equation proved be convenient for estimating individual VO_{2peak}.

Beutner et al. 2015 [52]	Females=56<br>Males=55<br>(Healthy)	22-79	YMCA step	Gas analyzer in maximal cycle test (Bruce protocol)	Correlation coefficient (r), linear regression, SEE	r=0.86 ($p<0.001$), SEE= 5.5 ml/kg/min	The YMCA step test is appropriate for estimating individual VO_{2peak}.
Lee et al. 2019 [60]	Female and Male=568<br>(Healthy)	20-66	YMCA step	Gas analyzer in maximal treadmill test (Bruce protocol)	Multiple linear regression, SEE, repeated ANOVA	r= -0.44 ($p<0.001$) between VO_{2max} and heart rate recovery.<br>Equation of YMCA step test: $R^2=0.60$ ($p<0.05$), SEE=4.76 ml/kg/min, mean difference=0.14±1.23 ml/kg/min ($p>0.05$).	The YMCA step test is accurate for estimating individual VO_{2peak}.
Hong et al. 2019 [59]	Females = 36<br>Males = 37<br>(Healthy)	32.3±8.9<br>29.4±9.5	YMCA step<br>Tecumseh step<br>6-minute walk	Gas analyzer in maximal treadmill test (Modified Bruce protocol)	Multiple linear regression, SEE, paired t-test, Bland-Altman method	r=0.5281 ($p<0.001$) between VO_{2max} and heart rate 30s in Tecumseh step test.<br>Equation of Tecumseh step test: $R^2=0.734$ ($p<0.05$), SEE=4.7 ml/kg/min, no mean difference ($p>0.05$).<br>r=0.682 ($p<0.001$) between VO_{2max} and heart rate 30s in YMCA step test.<br>Equation of YMCA step test: $R^2=0.722$ ($p<0.05$), SEE=4.7 ml/kg/min, no mean difference ($p>0.05$).<br>r=0.671 ($p<0.001$) between VO_{2max} and distance walked in 6-minute test.	The YMCA step, Tecumseh step and 6-minute walk tests are valid for estimating VO_{2max} in young healthy adults.

						Equation of 6-minute walk test: R^2=0.744 (p<0.05), SEE=4.6 ml/kg/min, no mean difference (p>0.05).	
Kieu et al. 2020 [43]	Female and Male=30<br>(Healthy)	19-35	YMCA step	Gas analyzer in maximal treadmill test (Bruce protocol)	Correlation coefficient paired t-test, Bland-Altman method	r=0.80 (p<0.001) between VO_{2max} measured and VO_{2ma} predicted.<br>Mean difference=2.26±5.47 ml/kg/min (p<0.001).	The YMCA step test, is valid for estimating VO_{2max} in young healthy adults.
Hansen et al. 2016 [55]	Females = 59<br>Males = 53<br>(Healthy)	18-75	Modified Harvard step	Gas analyzer in maximal cycle test	Correlation coefficient (r), linear regression, Bland-Altman method, ICC	r= -0.48 and 0.52 (p<0.001 for both) between VO_{2max} and heart rate and step test duration.<br>Equation of modified Harvard step test: R^2=0.78 (p<0.00)<br>Mean difference=0.0±0.4 L/min (p=1.000), between measured and predicted VO2max<br>ICC=0.94 (p<0.001) between measured and predicted VO2max.	The equation developed is valid to estimate VO_{2max} in healthy adults. However, only the test performance (heart rate) is not valid for estimating individual VO_{2max}.
Aadahl et al. 2012<br>[49]	Females=449<br>Males= 346<br>(Healthy)	47.0±8.0<br>46.7±8.4	Danish step	Gas analyzer in maximal cycle test (Watt-max protocol)	Correlation coefficient (r), Bland-Altman method	r=0.69 and 0.77 (p<0.001 both) between Danish step test and $VO2_{max}$, for male and female, respectively.<br>Mean difference=2.25±13.45 and 3.06±10.68 ml/kg/min, for male and female,	The Danish step test slightly overestimated VO_{2max} compared to the watt-max test, more so in female than in male.

						respectively.	
Kumar et al. 2012 [35]	Males = 19<br>(Healthy-sedentary)	20-30	Queen's College step	Gas analyzer in maximal cycle test	Correlation coefficient (r), linear regression, paired t-test, Bland-Altman method	r =0.034 ($p<0.05$) between Queen's College step test and $VO2_{max.}$<br>Mean difference=20±15.31 ml/kg/min.	Queen's College step test is not a valid test for this population.
Ricci et al. 2019<br>[41]	Female=31<br>(Obese with comorbidities and morbidly)	18-60	2-minute step	Gas analyzer in maximal treadmill test (Bruce protocol)	Correlation coefficient (r), stepwise multiple linear regression, student t-test, Bland-Altman method	r=0.55 ($p<0.01$) between up-and-down step cycles and $VO2_{max.}$<br>Equation of 2-minute step test: R^2=0.744 ($p<0.001$).<br>Mean difference=3.1±3.0 ml/kg/min ($p<0.05$).	The 2-minute step test underestimated $VO2_{max.}$
Cooper et al.<br>2005 [34]	Males=30<br>(Healthy active)	21.8±3.6	20-m shuttle run	Gas analyzer in maximal treadmill test	Paired t-test, Bland-Altman method,	Mean difference=1.8±6.3 ml/kg/min (p=0.004)	20-m shuttle run test is not a valid test for estimating individual VO_{2max} in active males.
Flouris et al. 2004 [33]	Males = 50<br>(Healthy)	21.6±1.6	20-m shuttle run<br>20-m square shuttle run	Gas analyzer in maximal treadmill test (Modified Bruce protocol)	linear regression, SEE, CV, one-way ANOVA	Equation of 20-m shuttle run test: r=0.61 ($p<0.05$), SEE=4.23 ml/kg/min, mean difference= -2.0±2.9 ml/kg/min ($p<0.001$), CV=7.9%.<br>Equation of 20-m square shuttle run test: r=0.88 ($p<0.001$), SEE=2.51 ml/kg/min, mean difference= -0.7±0.3	The 20-m square shuttle run test had a higher agreement with the gold standard laboratory test in predicting VO_{2max}.

						ml/kg/min ($p<0.001$), CV=6.0%	
Flouris et al. 2005 [46]	Males = 110<br>(Healthy)	21.6 ±2.5	20-m shuttle run	Gas analyzer in maximal treadmill test (Modified Bruce protocol)	Correlation coefficient (r), linear regression, Bland-Altman method, one-way ANOVA	r =0.94 and R^2=0.89 ($p<0.001$, for both), SEE=1.94 ml/kg/min between measured equation and predicted VO_{2max}.<br>Mean difference=0.1±0.6 ml/kg/min ($p>0.05$) between measured equation and predicted VO_{2max}.	The prediction model increases the efficacy of the 20-m shuttle run test for predicting VO_{2max}.
Metsios et al.<br>2008 [47]	Males = 74<br>(Healthy)	21±2	20-m shuttle run<br>20-m square shuttle run	Gas analyzer in maximal treadmill test (Modified Bruce protocol)	Correlation coefficient (r), 95% LoA, CV, one repeated measures ANOVA	Equation of 20-m shuttle run test: r=0.63 ($p<0.001$), 95%LoA=4.23±7.25 ($p<0.001$, CV=7.37%<br>Equation of 20-m square shuttle run test: r=0.95 ($p<0.001$), 95%LoA=-0.28±3.25 ($p>0.05$), CV=3.46%	The 20-m square shuttle run test is a valid and reproducible assessment tool and a more efficacious test for predicting VO_{2max} in healthy adult males compared to 20-m shuttle run test.
Kim et al. 2011 [48]	Females = 155<br>Males = 158<br>(Healthy)	28.3±4.1	20-m shuttle run	Gas analyzer in maximal treadmill test (Modified Bruce protocol)	Correlation coefficient (r), Bland-Altman method, one-way repeated measures ANOVA	r range from 0.86 to 0.95 (all $p<0.001$) between 20-m shuttle run test and VO_{2max}<br>Mean differences range from -0.54±6.23 to -2.94±6.55 (all $p<0.01$) between 20-m shuttle run test and VO_{2max}.	Equations based on the 20-m shuttle run test result, should not be applied for predicting VO_{2max} in Korean adults.
Jurio-Iriarte et al. 2017 [57]	Females = 75<br>Males = 181<br>(Overweight-	53.9±8.1	Incremental shuttle walk (15-level)	Gas analyzer in maximal cycle test	Correlation coefficient (r), linear regression, t-test, Bland-Altman method, SEE, E	r=0.72 and R^2=0.52 ($p<0.001$, for both), SEE= 4.35 ml/kg/min (19% E) between equation and	Modified shuttle walk test (15-level) does not accurately predict VO_{2max} in overweight/obese people

	obese/primary hypertension)					predicted VO_{2max}.<br>Mean difference=0.2±8.4 ml/kg/min (p=0.48) between equation and predicted VO_{2max}.	with primary hypertension.
Jurio-Iriarte et al. 2018 [58]	Females = 74<br>Males = 174<br>(Overweight-obese/ primary hypertension)	54.0±7.3	Incremental shuttle walk (15-level)	Gas analyzer in maximal cycle test	Correlation coefficient (r), linear regression, SEE, ICC, Bland-Altman method	r=0.72 and R^2=0.58 (p<0.001, for both), SEE= 4.9 ml/kg/min between measured and predicted VO_{2max}.<br>ICC=0.69 (0.34–0.82) between equation and predicted VO_{2max}.<br>Mean difference=3.7±9.0 ml/kg/min, SEE=4.35 ml/kg/min, between equation and predicted VO_{2max}.	Modified shuttle walk test (15-level) does not accurately predict VO_{2max} in overweight/obese people with primary hypertension.
Lima et al. 2019 [61]	Females = 54<br>(Sedentary)	18-45	Incremental shuttle walking (15-level)	Gas analyzer in maximal treadmill test	Correlation coefficient (r), stepwise multiple linear regression, paired t-test, Bland-Altman method,	r=0.57 (p<0.001) between VO_{2peak} measured and VO_{2peak} predicted.<br>R^2=0.363 (p<0.05) between prediction equation and $VO2_{peak}$ measured.<br>Mean difference= -0.14±9.27 ml/kg/min (p>0.05).	Incremental shuttle walking test is valid to assess cardiorespiratory fitness in sedentary females.
Oja et al. 1991 [24]	Females = 80<br>Males = 79<br>(Healthy)	20-65	2-km walk	Gas analyzer in maximal treadmill test (Modified Pennsylvania State	Correlation coefficient (r), multivariate linear regression; SEE	r= -0.65 and -0.74 (p<0.001, for both), between walking time VO_{2max}, for male and female, respectively.	2-km walk test is an accurate alternative for predicting VO_{2max} in healthy adults.

				University protocol)		R^2=0.75, SEE=5.1 ml/kg/min (p<0.05) for prediction model in male.<br>R^2=0.73, SEE=3.3 ml/kg/min (p<0.05) for prediction model in female.	
Laukkanen et al. 1992 [25]	Females = 32<br>Males = 36<br>(Overweight/obese)	20-65	2-km walk	Gas analyzer in maximal treadmill test	Correlation coefficient (r), E	r= -0.49 (p<0.01) and -0.72 (p<0.001) between walking time and VO_{2max}, for male and female, respectively.<br>r= 0.75 and 0.77 (p<0.001) for prediction model in male and female, respectively.<br>E=6.9 ml/kg/min in male and 3.2 ml/kg/min in female.	2-km walk test is valid predicting VO_{2max} in overweight/obese individuals, although slightly underestimate in owerweight/obese men.
Laukkanen et al. 1993 [27]	Females = 32<br>Males = 79<br>(Moderate active and highly active healthy)	35-45	2-km walk	Gas analyzer in maximal treadmill test	Correlation coefficient (r), linear regression, TE	r= -0.73 (p<0.05), -0.52 (p<0.05), and -0.31 (p<0.05), between walking time and VO_{2max}, for moderate active male and female, and highly active male, respectively.<br>r=0.80 (p<0.05), TE=5.7 ml/kg/min for prediction model in moderate active male.<br>r=0.55 (p<0.05), TE=5.0 ml/kg/min for prediction model in moderate active female.<br>r=0.60 (p<0.05), TE=7.7	2-km walk test is a reasonably valid field test for estimating the cardiorespiratory fitness of healthy men and women, except for people with very high maximal aerobic power.

						ml/kg/min for prediction model in moderate active male.	
Laukkanen et al. 2000 [45]	Females = 61<br>Males = 55<br>(Healthy)	30-55	2-km walk	Gas analyzer in maximal treadmill test	Two-way ANOVA, TE	Mean difference=0.9±4.4 ml/kg/min, TE=4.5 ml/kg/min in male.<br>Mean difference=2.2±3.5 ml/kg/min, TE=4.1 ml/kg/min in female.	2-km walk test has a reasonably accuracy to predict VO_{2max} in untrained healthy adults.
Kline et al. 1987 [23]	Females=178<br>Males=165<br>(Healthy)	30-69	1-mile walk	Gas analyzer in maximal treadmill test	Correlation coefficient (r), multiple linear regression, SEE	r=0.64 ($p<0.05$) between time test and VO_{2max}.<br>r=0.88, SEE=5.0 l/min ($p<0.05$) for prediction model.	1-mile walk test is valid to assess VO2max in this population.
Dolgener et al. 1994 [44]	Females=145<br>Males=129<br>(Healthy)	19.3±2.32<br>19.4±3.08	1-mile walk	Gas analyzer in maximal treadmill test	Correlation coefficient, (r), multiple linear regression, SEE, E	r=0.69 ($p<0.05$), SEE=5.5 ml/kg/min, E=13.26 between Kline´s equation and $VO2_{max}$.<br>New predicted equation (l/min): r=0.86 ($p<0.05$), SEE=0.383, E=0.953.<br>New predicted equation (ml/kg/min): r=0.69 ($p<0.05$), SEE= 5.500, E=13.26.	1-mile walk test over predicts VO_{2max} in college students and should not be used with this population.
George et al. 1998 [29]	Females = 59<br>Males = 39<br>(Healthy)	18-29	1-mile walk	Gas analyzer in maximal treadmill test	Correlation coefficients (r), SEE, E, paired t-test,	Kline´s equation:<br>r=0.84 ($p<0.05$), SEE=3.61ml/kg/min, E=6.16, overpredicted ±4.98	The Dolgener´s equation seems to be more accurate that Kline´s equation to predict VO_{2max}.

						ml/kg/min ($p<0.05$).<br>Dolgener´s equation:<br>r=0.85 ($p<0.05$), SEE=3.48 ml/kg/min, E=3.74, underestimated ±1.24 ml/kg/min ($p<0.05$).	
Seneli et al. 2013 [36]	Females = 10<br>Males = 13<br>(Healthy)	19-44	1-mile walk	Gas analyzer in maximal treadmill test	Correlation coefficient (r), Bland-Altman method, one-way repeated measures ANOVA	r range from 0.71 to 0.84 (all $p<0.05$) between equations and VO_{2max}.<br>Mean differences range from 2.360 to 9.131 ml/kg/min (all $p<0.001$, except for 2.360 where $p=0.064$).	Despite high correlations, Kline´s and Dolgener´s equations of 1-mile walk test underestimate VO_{2max}. It would be beneficial if a new equation were created specifically for the curved treadmill.
Lunt et al. 2013 [51]	Females = 44<br>Males = 162<br>(Healthy)	18-39	1-mile walk	Gas analyzer in maximal treadmill test	Stepwise linear regression, SEE, one-way repeated measures ANOVA,	$R^2=0.68$ ($p<0.05$), SEE=4.6 ml/kg/min, -0.5%, mean difference=1.2 ml/kg/min ($p>0.05$), between new equation and VO_{2peak}.	The new equation derived from this study gave the most accurate estimate of VO_{2peak}.
Greenhalgh et al. 2001 [31]	Females = 24<br>Males = 21<br>(Healthy)	18-29	1-mile walk<br>1/4-mile walk	Gas analyzer in maximal treadmill test	Correlation coefficient (r), SEE, TE, % ±4.5 ml/kg/min	r=0.81 ($p<0.05$), SEE=4.33 ml/kg/min, TE=4.8, % 67.5 and r=0.84 ($p<0.05$), SEE=4.03 ml/kg/min, TE=4.12, % 75.7 between Kline´s equation and 1/4-mile walk test and 1-mile walk test, respectively.<br>r=0.84 ($p<0.05$), SEE=4.03 ml/kg/min, TE=7.07, % 43.52 and r=0.85 ($p<0.05$), SEE=3.93 ml/kg/min,	The 1/4-mile walk test predicts VO_{2max} with similar accuracy as the1-mile walk test.

						TE=47.93, % 18.9 between Dolgener´s equation and 1/4-mile walk test and 1-mile walk test, respectively.	
Larsen et al. 2002 [32]	Female = 47<br>Male = 52<br>(Healthy)	18-26	1.5-mile run/walk	Gas analyzer in maximal treadmill test	Multiple linear regression, SEE	R^2=0.86 ($p<0.05$), SEE=3.37 ml/kg/min, between new equation and VO_{2max}.	1.5-mile test is valid without measuring heart rate.
McNaughton et al. 1998 [30]	Males= 32<br>(Active healthy)	20±0.3	20-m shuttle run<br>12-minute run<br>1.5-mile run<br>Treadmill jogging	Gas analyzer in maximal treadmill test	Correlation coefficient (r), SE, one-way ANOVA	r=0.82 ($p<0.05$), SE=1.0, mean difference=1.62 ml/kg/min ($p>0.05$), between 20-m shuttle run test and VO_{2max}.<br>r=0.87 ($p<0.05$), SE=0.7, mean difference= -1.02 ml/kg/min ($p>0.05$), between 12-minute run test and VO_{2max}.<br>r=0.87 ($p<0.05$), SE=1.2, mean difference=0.74 ml/kg/min ($p>0.05$), between 1.5-mile run and VO_{2max}.<br>r=0.50 ($p<0.05$), SE=1.5, mean difference=5.58 ml/kg/min ($p>0.05$), between treadmill jogging test and VO_{2max}.	The 12-minute run test is the best predictor of VO_{2max} in young adult males with active to trained levels of fitness. The submaximal treadmill jogging test has low-moderate validity to predict VO_{2max}.
George et al. 1993 [28]	Females = 45<br>Males = 84<br>(Healthy-	18-29	Submaximal single-stage treadmill	Gas analyzer in maximal treadmill test	Multiple linear regression, SEE	r=0.84, ($p<0.001$), SEE=3.2 ml/kg/min between prediction equation and	Submaximal single-stage treadmill jogging test is valid for estimating VO_{2max}.

	active)		jogging			VO_{2max}.	
Cao et al. 2013 [50]	Females=140<br>Males=143<br>(Healthy)	20-69	3-minute walk	Gas analyzer in maximal cycle test	Multiple linear regression, SEE, %SEE	r =0.55 (p<0.001) between time of 3-minute walk test and VO_{2max}.<br>Best equation %Fat: R^2=0.70 (p<0.001), SEE=4.565 ml/kg/min, % SEE=13.316.	The 3-minute walk test is a valid tool for estimating VO_{2max} in Japanese adult men and women.
Di Thommazo-Luporini et al. 2015 [53]	Females=51<br>(Sedentary obese)	20-45	6-minute walk<br>Incremental shuttle walking	Gas analyzer in maximal treadmill test (Bruce protocol)	Correlation coefficient (r), linear regression, Bland-Altman method	r=0.70 and 0.62 (all p<0.001) between VO_{2max} and 6-minute walk and incremental shuttle walking tests<br>There was agreement between Bruce protocol and both the incremental shuttle walking test: VO_{2max}=6.1 (CI: 4.9–7.3), and heart rate=36.2 (CI: 32.1–40.3); and 6-minute walk test: VO_{2max}=6.9 (CI: 5.7–8.1), and heart rate=37.0 (CI: 33.3–40.7).	6-minute walk and incremental shuttle walking tests are valid to assess cardiorespiratory fitness in obese females.
Di Thommazo-Luporini et al. 2016 [54]	Females=56<br>(Sedentary obese)	35±7	6-minute walk	Gas analyzer in maximal treadmill test (Bruce protocol test)	Correlation coefficient (r), stepwise multiple linear regression, Bland-Altman method	r=0.56 (p<0.001) between VO_{2max} and up and down step cycles 6-minute walk.<br>Mean difference: 5.1±3.6 ml/kg/min.	6-minute walk test is valid to assess cardiorespiratory fitness in obese females.
Manttari et al. 2018 [40]	Females = 36<br>Males = 39	19-75	6-minute walk	Gas analyzer in maximal treadmill test	Linear regression, SEE, Bland-Altman method	R^2=0.85(p<0.001) and SEE=3.9 ml/kg/min, between prediction equation	6-minute walk test is an accurate and valid tool for estimating VO_{2max}.

	(Healthy)			Douglas bag methods		and $VO2_{max}$, in male.<br>R^2=0.90 ($p<0.001$) and SEE=3.1 ml/kg/min, between prediction equation and $VO2_{max}$, in female.<br>Mean difference: 0±5.4 ml/kg/min.	
Jamnick et al. 2016 [56]	Females = 26<br>Males = 31<br>(Healthy)	18-39	YMCA cycle<br>Mankato submaximal exercise	Gas analyzer in maximal treadmill test	Linear regression, ICC, SEE, TE, CV, one-way repeated measures ANOVA,	r=0.646 ($p<0.05$), ICC=0.271, SEE=6.91 ml/kg/min, TE=5.57, CV=15.17%, mean difference=3.96 ml/kg/min ($p<0.01$), between YMCA test and VO_{2max}.<br>r=0.72 ($p<0.05$), ICC=0.722, SEE=6.20 ml/kg/min, TE=4.62, CV=11.28%, mean difference= -1.54 ml/kg/min ($p>0.05$), between Mankato submaximal exercise test and VO_{2max}.	The Mankato submaximal exercise test yield better estimates of VO_{2max}. The YMCA test underestimates VO_{2max}
Siconolfi et al. 1982 [22]	Females = 28<br>Males = 35<br>(Healthy)	20-70	Modified Astrand-Ryhming	Gas analyzer in maximal cycle test	Linear regression, SEE, paired t-test	r=0.86 ($p<0.05$), SEE=0.359 l/min, mean difference=0.07 ml/kg/min ($p>0.05$), between prediction equation and $VO2_{max}$ in males.<br>r=0.97 ($p<0.05$), SEE=0.199 l/min, mean difference= -0.04 ml/kg/min ($p>0.05$), between prediction equation and $VO2_{max}$ in females.	Modified Astrand-Ryhming test is valid for estimating VO_{2max}.

Bonet et al. 2020 [42]	Females = 42 (Healthy-active)	35-45	The University Montreal track (UM-track)	Gas analyzer in treadmill performing UM-track test	Correlation coefficient (r), linear regression, paired t-test, Cohen's d effect size, Bland-Altman method	r =0.93 (p<0.001) between time in UM-track test and UM-treadmill test.<br>r =0.71 (p<0.001) between time in UM-track test and $VO2_{max}$.<br>Mean difference=0.025±7.445 ml/kg/min (p>0.05), Cohen's d<0.5.	UM- track test valid for estimating VO_{2max}. in middle-aged active women.
Guo et al. 2018 [39]	Females = 22<br>Males = 18<br>(Healthy)	18-64	Ruffier	Gas analyzer in treadmill (modified Balke protocol)	Multiple linear regression, RMSE, sensitivity and specificity, kappa	R^2=0.637 (p<0.05), RMSE=0.517, sensitivity=0.79 and specificity=0.56, *k*= 0.60 between prediction equation and $VO2_{max}$.	Ruffier test has moderate validity for estimating $VO2_{max}$.
Musculoskeletal fitness (isometric)							
Shechtman et al. 2005 [97]	Females = 50<br>Males = 50<br>(Healthy)	20-40	DynEx dynamometer	Known weights<br>Jamar dynamometer	Correlation coefficient (r), measurement error (%), repeated measures ANCOVA	r =0.99 (p<0.05) between DynEx and Jamar dynamometers.<br>Average measurement error =1.63% and 7.74% for DynEx and Jamar dynamometer, respectively, in relation known weights.<br>Significant differences in grip strength scores between DynEx and Jamar dynamometers (F=6.222, p=0.014).	The DynEx dynamometer is valid for assessing grip strength.

España-Romero et al. 2010 [102]	NA	NA	TKK, DynEx and Jamar dynamometers	Known weights	Bland-Altman method	Negative systematic error for the Jamar and DynEx dynamometer (-1.92±1.92 and -1.43±3.56 kg, respectively, ($p<0.05$). Positive systematic bias for the TKK dynamometer (0.49±1.32 kg, $p<0.05$).	TKK dynamometer is the most appropriate dynamometer to assess grip strength.
Cadenas-Sánchez et al. 2016 [103]	NA	NA	TKKdynamometers (old and new digitals and analog)	Known weights	One-sample t-test, Bland-Altman method	Negative systematic error for the new digital (-2.64 to -2.02 kg), new analogic (-2.15 kg to -1.51 kg), old digital and analogic (-0.94 kg and -2.29 kg, respectively). All $p<0.001$.	TKK dynamometer is valid to assess grip strength.
Kolimechkov et al. 2020 [104]	NA	NA	TKK and DynEx and dynamometers	Known weights	Paired t-test, Bland-Altman method	Non-significant systematic error of -0.20±0.62 kg ($p>0.05$) for the TKK, and a significant systemic error of -0.42±0.46 kg ($p<0.001$) for the DynEX dynamometer.	TKK dynamometer showed to be more valid than DynEX dynamometer.
Musculoskeletal fitness (endurance)							
Mannion et al. 1994 [95]	Female = 208<br>Male = 21<br>(Healthy)	26.9±4.2	Biering-Sørensen	Electromyography	Linear regression	R^2=0.69 (p=0.001), between time endurance and changes in median frequency	The Biering-Sørensen test is valid for measuring back muscle fatigue.
Kankaanpää et al. 1998 [96]	Female = 153	38.9±7.6	Biering-	Electromyography	Correlation coefficients (r), multiple linear	r ranges from 0.60 to 0.71 ($p<0.05$), between NMF_{slope}	The validity of this test to assess lumbar muscle

	Male = 100<br><br>(Healthy)		Sørensen		regression	and the endurance time.<br><br>BMI contributed most of the variance in women: R^2=0.29 (p<0.001) and BMI and age in men: R^2=0.12 (p<0.05).	endurance is acceptable.
Coorevits et al. 2008 [98]	Female = 13<br><br>Male = 7<br><br>(Healthy-active)	23.5±1.1<br><br>32.9±14.3	Biering-Sørensen	Electromyography	Correlation coefficients (r), multiple stepwise linear regression, two-way ANOVA	r ranges from 0.46 to 0.71 (p<0.05), between NMF_{slope} and the endurance time.<br><br>The most predictive muscle was iliocostalis lumborum pars thoracis: R^2=0.477 (p<0.001).<br><br>Difference between both methods to estimated end point test (±16 sec., p=0.22); but highly correlated: r ranges 0.94 to 0.98 (p<0.01).	The Biering-Sørensen test is valid for measuring back muscle fatigue.
De Blaiser et al. 2018 [100]	Female = 15<br><br>Male = 14<br><br>(Healthy-active)	25.5±2.1	Prone bridging	Electromyography	Correlation coefficients (r), multiple backward linear regression, one-way repeated measures ANOVA,	r=0.59 (p<0.01) between NMF_{slope} and the endurance time.<br><br>Endurance time is the best predictor: R^2=0.261 (p<0.05).<br><br>NMF_{slope} values showed non-significant differences between the different muscles (p>0.05).	The prone bridging test is valid for evaluating core muscle fatigue.

Musculoskeletal fitness (explosive)							
Bui et al. 2015 [99]	Females=18<br>Males=23<br>(Healthy)	19-40	Sargent jump	Contact mat<br>Optical system	Linear regression, repeated measures ANOVA, Bland-Altman method	R^2=0.8 (p<0.05) between contact mat and Sargent jump.<br>R^2=0.76 (p<0.05) between optical sensor and Sargent jump.<br>Positive systematic error=4.4±5.1 and 4.8±5.7 cm (p<0.001 for both) between the Sargent jump with contact mat and optical sensor, respectively.	Sargent jump test overestimates the height of a vertical jump and its accuracy is reduced as jump height increase.
Musculoskeletal fitness (flexibility)							
Kawano et al. 2010 [101]	Females = 100<br>Males = 100<br>(Healthy-active)	18-25	Sit-and-reach<br>(Hip joint angle)	Angular kinematic analysis	Correlation coefficient (r), one-way repeated measures ANOVA, 95%CI	r=0.48 (p<0.05) between sit-and- reach and kinematic analysis with ankle dorsiflexed.<br>*r*=0.44 (p<0.05) between sit-and- reach and kinematic analysis with ankle plantarflexed.<br>The hip joint angle values for men presented significant statistical dif-ferences when comparing dorsiflexed (=83.0º) with plantarflexed (87.3º), 95% CI of mean difference (3.6-4.9; p<0.0001).	Sit-and- reach test is not valid to assess hip joint angle.

These were similar to those found for women (dorsiflexed =107.9º; plantarflexed =112.9º), 95% CI of mean difference (3.5-4.9; $p<0.001$).

ANOVA indicates analysis of variance; ANCOVA, analysis of covariance; BMI, body mass index; CI, coefficient of interval; CV, coefficient of variation; E, error; ICC, intraclass correlation coefficient; MANOVA, multiple analysis of variance; NMF_{slope}, normalized median frequency slope; RM, repetition maximum; RMSE, root mean square error; SEE, standard error estimate; TE, total error; VO_{2max}, maximum oxygen uptake; VO_{2peak}, peak oxygen uptake.

REFERENCES

1. Mayorga-Vega, D.; Merino-Marban, R.; Viciana, J., Criterion-related validity of sit-and-reach tests for estimating hamstring and lumbar extensibility: a meta-analysis. *Journal of Sports Science & Medicine* **2014,** *13* (1), 1-14.
2. Mayorga-Vega, D.; Viciana, J.; Cocca, A.; Merino-Marban, R., Criterion-related validity of toe-touch test for estimating hamstring extensibility: A metaanalysis. *J Hum Sport Exerc* **2014,** *9* (1), 188-200.
3. Bennett, H.; Parfitt, G.; Davison, K.; Eston, R., Validity of submaximal step tests to estimate maximal oxygen uptake in healthy adults. *Sports Medicine* **2016,** *46* (5), 737-750.
4. Mayorga-Vega, D.; Aguilar-Soto, P.; Viciana, J., Criterion-related validity of the 20-m shuttle run test for estimating cardiorespiratory fitness: a meta-analysis. *Journal of Sports Science & Medicine* **2015,** *14* (3), 536-547.
5. Mayorga-Vega, D.; Bocanegra-Parrilla, R.; Ornelas, M.; Viciana, J., Criterion-related validity of the distance- and time-based walk/run field tests for estimating cardiorespiratory fitness: a systematic review and Meta-analysis. *PloS One* **2016,** *11* (3), e0151671-e0151671.
6. Leger, L.; Boucher, R., An indirect continuous running multistage field test: the Universite de Montreal track test. *Can. J. Appl. Sport. Sci* **1980,** *5*, 77-84.
7. Santa Maria, D.; Kinnear, G. R.; Kearney, J. T.; Martin, T. P., The objectivity, reliability, and validity of the OSU step test for college males. *Research Quarterly* **1976,** *47* (3), 445-452.
8. Siconolfi, S. F.; Garber, C. E.; Lasater, T. M.; Carleton, R. A., A simple, valid step test for estimating maximal oxygen uptake in epidemiologic studies. *American Journal of Epidemiology* **1985,** *121* (3), 382-390.
9. Ritchie, C.; Trost, S. G.; Brown, W.; Armit, C., Reliability and validity of physical fitness field tests for adults aged 55 to 70 years. *Journal of Science and Medicine in Sport* **2005,** *8* (1), 61-70.
10. Azmi, S. H.; Sulaiman, N., Validity of selected cardiovascular field-based test among Malaysian healthy female adult. *J Fundam Appl Sci* **2017,** *9*, 1227-1235.
11. McGawley, K., The Reliability and Validity of a Four-Minute Running Time-Trial in Assessing VO2max and Performance. *Frontiers in Physiology* **2017,** *8*, 270.
12. Giacomantonio, N.; Morrison, P.; Rasmussen, R.; MacKay-Lyons, M. J., Reliability and validity of the 6-minutestep test for clinical assessment of cardiorespiratory fitness in people at risk of cardiovascular disease. *Journal of Strength and Conditioning Research* **2020,** *34* (5), 1376-1382.
13. Leger, L. A.; Mercier, D.; Gadoury, C.; Lambert, J., The multistage 20 metre shuttle run test for aerobic fitness. *JOURNAL OF SPORTS SCIENCES* **1988,** *6* (2), 93-101.

14. Flouris, A. D.; Metsios, G. S.; Famisis, K.; Geladas, N.; Koutedakis, Y., Prediction of VO2max from a new field test based on portable indirect calorimetry. *J Sci Med Spor* **2010,** *13* (1), 70-73.
15. Gabriel, K. K. P.; Rankin, R. L.; Lee, C.; Charlton, M. E.; Swan, P. D.; Ainsworth, B. E.; Pettee Gabriel, K. K.; Rankin, R. L.; Lee, C.; Charlton, M. E.; Swan, P. D.; Ainsworth, B. E., Test-retest reliability and validity of the 400-meter walk test in healthy, middle-aged women. *J Phys Act Health* **2010,** *7* (5), 649-657.
16. Hansen, D.; Jacobs, N.; Bex, S.; D'Haene, G.; Dendale, P.; Claes, N., Are fixed-rate step tests medically safe for assessing physical fitness? *European Journal of Applied Physiology* **2011,** *111* (10), 2593-2599.
17. Burr, J. F.; Bredin, S. S.; Faktor, M. D.; Warburton, D. E., The 6-minute walk test as a predictor of objectively measured aerobic fitness in healthy working-aged adults. *Physician and Sportsmedicine* **2011,** *39* (2), 133-9.
18. Mikawa, K.; Senjyu, H., Development of a field test for evaluating aerobic fitness in middle-aged adults: Validity of a 15-m Incremental Shuttle Walk and Run Test. *Journal of Sports Science & Medicine* **2011,** *10* (4), 712-717.
19. Arcuri, J. F.; Borghi-Silva, A.; Labadessa, I. G.; Sentanin, A. C.; Candolo, C.; Pires Di Lorenzo, V. A., Validity and reliability of the 6-minute step test in healthy individuals: a cross-sectional study. *Clinical Journal of Sport Medicine* **2016,** *26* (1), 69-75.
20. Jurgensen, S. P.; Trimer, R.; Dourado, V. Z.; Di Thommazo-Luporini, L.; Bonjorno-Junior, J. C.; Oliveira, C. R.; Arena, R.; Mendes, R. G.; Borghi-Silva, A., Shuttle walking test in obese women: test-retest reliability and concurrent validity with peak oxygen uptake. *Clinical Physiology and Functional Imaging* **2015,** *35* (2), 120-126.
21. Lerche, L.; Olsen, A.; Petersen, K. E. N.; Rostgaard-Hansen, A. L.; Dragsted, L. O.; Nordsborg, N. B.; Tjonneland, A.; Halkjaer, J., Validity of physical activity and cardiorespiratory fitness in the Danish cohort "Diet, Cancer and Health-Next Generations". *Scandinavian Journal of Medicine & Science in Sports* **2017,** *27* (12), 1864-1872.
22. Siconolfi, S. F.; Cullinane, E. M.; Carleton, R. A.; Thompson, P. D., Assessing VO2max in epidemiologic studies: modification of the Astrand-Rhyming test. *Medicine and Science in Sports and Exercise* **1982,** *14* (5), 335-338.
23. Kline, C.; Porcari, J. P.; Hintermeister, R.; Freedson, P. S.; Ward, A.; McCarron, R. F.; Ross, J.; Rippe, J., Estimation of from a one-mile track walk, gender, age and body weight. *Med. Sports Exerc* **1987,** *19*, 253-259.
24. Oja, P.; Laukkanen, R.; Pasanen, M.; Tyry, T.; Vuori, I., A 2-km walking test for assessing the cardiorespiratory fitness of healthy adults. *Int J Sports Med* **1991,** *12* (4), 356-62.
25. Laukkanen, R.; Oja, P.; Pasanen, M.; Vuori, I., Validity of a two kilometre walking test for estimating maximal aerobic power in overweight adults. *Int J Obes Relat Metab Disord* **1992,** *16* (4), 263-8.
26. Weller, I. M.; Thomas, S. G.; Cox, M. H.; Corey, P. N., A study to validate the Canadian Aerobic Fitness Test. *Can J Public Health* **1992,** *83* (2), 120-4.

27. Laukkanen, R. M. T.; Oja, P.; Pasanen, M. E.; Vuori, I. M., Criterion validitv of a two-kilometer walking test for predicting the maximal oxygen uptake of moderately to highly active middle-aged adults. *Scandinavian Journal of Medicine and Science in Sports* **1993,** (3), 267-272.
28. George, J. D.; Vehrs, P. R.; Allsen, P. E.; Fellingham, G. W.; Fisher, A. G., Development of a submaximal treadmill jogging test for fit college-aged individuals. *Medicine and Science in Sports and Exercise* **1993,** *25* (5), 643-647.
29. George, J. D.; Fellingham, G. W.; Fisher, A. G., A modified version of the Rockport Fitness Walking Test for college men and women. *Research Quarterly for Exercise and Sport* **1998,** *69* (2), 205-209.
30. McNaughton, L.; Hall, P.; Cooley, D., Validation of several methods of estimating maximal oxygen uptake in young men. *Perceptual and Motor Skills* **1998,** *87* (2), 575-584.
31. Greenhalgh, H. A.; George, J. D.; Hager, R. L., Cross-validation of a quarter-mile walk test using two VO2 max regression models. *Meas Phys Educ Exerc Sci* **2001,** *5* (3), 139-151.
32. Larsen, G. E.; George, J. D.; Alexander, J. L.; Fellingham, G. W.; Aldana, S. G.; Parcell, A. C., Prediction of maximum oxygen consumption from walking, jogging, or running. *Research Quarterly for Exercise and Sport* **2002,** *73* (1), 66-72.
33. Flouris, A. D.; Koutedakis, Y.; Nevill, A.; Metsios, G. S.; Tsiotra, G.; Parasiris, Y., Enhancing specificity in proxy-design for the assessment of bioenergetics. *Journal of Science and Medicine in Sport* **2004,** *7* (2), 197-204.
34. Cooper, S. M.; Baker, J. S.; Tong, R. J.; Roberts, E.; Hanford, M., The repeatability and criterion related validity of the 20 m multistage fitness test as a predictor of maximal oxygen uptake in active young men. *Br J Sports Med* **2005,** *39* (4), e19.
35. Kumar, S. K.; Khare, P.; Jaryal, A. K.; Talwar, A., Validity of heart rate based nomogram fors estimation of maximum oxygen uptake in Indian population. *Indian Journal of Physiology and Pharmacology* **2012,** *56* (3), 279-283.
36. Seneli, R. M.; Ebersole, K. T.; O'Connor, K. M.; Snyder, A. C., Estimated VO2max from the Rockport Walk Test on a Nonmotorized Curved Treadmill. *Journal of Strength and Conditioning Research* **2013,** *27* (12), 3495-3505.
37. Carvalho, L. P.; Di Thommazo-Luporini, L.; Aubertin-Leheudre, M.; Bonjorno Junior, J. C.; de Oliveira, C. R.; Luporini, R. L.; Mendes, R. G.; Lopes Zangrando, K. T.; Trimer, R.; Arena, R.; Borghi-Silva, A., Prediction of cardiorespiratory fitness by the six-minute step test and its association with muscle strength and power in sedentary obese and lean young women: a cross-sectional study. *PloS One* **2015,** *10* (12), e0145960.
38. Teren, A.; Zachariae, S.; Beutner, F.; Ubrich, R.; Sandri, M.; Engel, C.; Loeffler, M.; Gielen, S., Incremental value of veterans specific activity questionnaire and the ymca-step test for the assessment of cardiorespiratory fitness in population-based studies. *Eur J Prev Cardiol* **2016,** *23* (11), 1221-1227.
39. Guo, Y.; Bian, J.; Li, Q.; Leavitt, T.; Rosenberg, E. I.; Buford, T. W.; Smith, M. D.; Vincent, H. K.; Modave, F., A 3-minute test of cardiorespiratory fitness for use in primary care clinics. *PloS One* **2018,** *13* (7), e0201598.

40. Manttari, A.; Suni, J.; Sievanen, H.; Husu, P.; Vaha-Ypya, H.; Valkeinen, H.; Tokola, K.; Vasankari, T., Six-minute walk test: a tool for predicting maximal aerobic power (VO2 max) in healthy adults. *Clinical Physiology and Functional Imaging* **2018**.
41. Ricci, P. A.; Cabiddu, R.; Jürgensen, S. P.; André, L. D.; Oliveira, C. R.; Di Thommazo-Luporini, L.; Ortega, F. P.; Borghi-Silva, A., Validation of the two-minute step test in obese with comorbibities and morbidly obese patients. *Brazilian Journal of Medical and Biological Research* **2019,** *52* (9), e8402.
42. Bonet, J. B.; Magalhaes, J.; Viscor, G.; Pages, T.; Javierre, C. F.; Torrella, J. R., A field tool for the aerobic power evaluation of middle-aged female recreational runners. *Women and Health* **2020**.
43. Kieu, N. T. V.; Jung, S. J.; Shin, S. W.; Jung, H. W.; Jung, E. S.; Won, Y. H.; Kim, Y. G.; Chae, S. W., The validity of the YMCA 3-minute step test for estimating maximal oxygen uptake in healthy Korean and Vietnamese adults. *J Lifestyle Med* **2020,** *10* (1), 21-29.
44. Dolgener, F. A.; Hensley, L. D.; Marsh, J. J.; Fjelstul, J. K., Validation of the Rockport Fitness Walking Test in college males and females. *Research Quarterly for Exercise and Sport* **1994,** *65* (2), 152-158.
45. Laukkanen, R. M. T.; Kukkonen-Harjula, T. K.; Oja, P.; Pasanen, M. E.; Vuori, I. M., Prediction of change in maximal aerobic power by the 2-km walk test after walking training in middle-aged adults. *International Journal of Sports Medicine* **2000,** *21* (2), 113-116.
46. Flouris, A. D.; Metsios, G. S.; Koutedakis, Y., Enhancing the efficacy of the 20 m multistage shuttle run test. *British Journal of Sports Medicine* **2005,** *39* (3), 166-170.
47. Metsios, G. S.; Flouris, A. D.; Koutedakis, Y.; Nevill, A., Criterion-related validity and test-retest reliability of the 20m square shuttle test. *Journal of Science and Medicine in Sport* **2008,** *11* (2), 214-217.
48. Kim, J.; Jung, S. H.; Cho, H. C., Validity and Reliability of Shuttle-Run Test in Korean Adults. *International Journal of Sports Medicine* **2011,** *32* (8), 580-585.
49. Aadahl, M.; Zacho, M.; Linneberg, A.; Thuesen, B. H.; Jorgensen, T., Comparison of the Danish step test and the watt-max test for estimation of maximal oxygen uptake: the Health2008 study. *Eur J Prev Cardiol* **2013,** *20* (6), 1088-1094.
50. Cao, Z.-B.; Miyatake, N.; Aoyama, T.; Higuchi, M.; Tabata, I., Prediction of maximal oxygen uptake from a 3-minute walk based on gender, age, and body composition. *J Phys Act Health* **2013,** *10* (2), 280-287.
51. Lunt, H.; Roiz De Sa, D.; Roiz De Sa, J.; Allsopp, A., Validation of one-mile walk equations for the estimation of aerobic fitness in British military personnel under the age of 40 years. *Military Medicine* **2013,** *178* (7), 753-759.
52. Beutner, F.; Ubrich, R.; Zachariae, S.; Engel, C.; Sandri, M.; Teren, A.; Gielen, S., Validation of a brief step-test protocol for estimation of peak oxygen uptake. *Eur J Prev Cardiol* **2015,** *22* (4), 503-512.

53. Di Thommazo-Luporini, L.; Pinheiro Carvalho, L.; Luporini, R.; Trimer, R.; Falasco Pantoni, C. B.; Catai, A. M.; Arena, R.; Borghi-Silva, A., The six-minute step test as a predictor of cardiorespiratory fitness in obese women. *European Journal of Physical and Rehabilitation Medicine* **2015,** *51* (6), 793-802.
54. Di Thommazo-Luporini, L.; Carvalho, L. P.; Luporini, R. L.; Trimer, R.; Falasco Pantoni, C. B.; Martinez, A. F.; Catai, A. M.; Arena, R.; Borghi-Silva, A., Are cardiovascular and metabolic responses to field walking tests interchangeable and obesity-dependent? *Disability and Rehabilitation* **2016,** *38* (18), 1820-1829.
55. Hansen, D.; Jacobs, N.; Thijs, H.; Dendale, P.; Claes, N., Validation of a single-stage fixed-rate step test for the prediction of maximal oxygen uptake in healthy adults. *Clinical Physiology and Functional Imaging* **2016,** *36* (5), 401-406.
56. Jamnick, N. A.; By, S.; Pettitt, C. D.; Pettitt, R. W., Comparison of the YMCA and a custom submaximal exercise test for determining Vo2max. *Medicine and Science in Sports and Exercise* **2016,** *48* (2), 254-259.
57. Jurio-Iriarte, B.; Gorostegi-Anduaga, I.; Rodrigo Aispuru, G.; Perez-Asenjo, J.; Brubaker, P. H.; Maldonado-Martin, S., Association between Modified Shuttle Walk Test and cardiorespiratory fitness in overweight/obese adults with primary hypertension: EXERDIET-HTA study. *Journal of the American Society of Hypertension* **2017,** *11* (4), 186-195.
58. Jurio-Iriarte, B.; Brubaker, P. H.; Gorostegi-Anduaga, I.; Corres, P.; Martinez Aguirre-Betolaza, A.; Maldonado-Martin, S., Validity of the modified shuttle walk test to assess cardiorespiratory fitness after exercise intervention in overweight/obese adults with primary hypertension. *Clinical and Experimental Hypertension* **2018**, 1-6.
59. Hong, S. H.; Yang, H. I.; Kim, D. I.; Gonzales, T. I.; Brage, S.; Jeon, J. Y., Validation of submaximal step tests and the 6-min walk test for predicting maximal oxygen consumption in young and healthy participants. *International Journal of Environmental Research and Public Health* **2019,** *16* (23).
60. Lee, O.; Lee, S.; Kang, M.; Mun, J.; Chung, J., Prediction of maximal oxygen consumption using the Young Men's Christian Association-step test in Korean adults. *European Journal of Applied Physiology* **2019,** *119* (5), 1245-1252.
61. Lima, L. P.; Leite, H. R.; Matos, M. A.; Neves, C. D. C.; Lage, V.; Silva, G. P. D.; Lopes, G. S.; Chaves, M. G. A.; Santos, J. N. V.; Camargos, A. C. R.; Figueiredo, P. H. S.; Lacerda, A. C. R.; Mendonça, V. A., Cardiorespiratory fitness assessment and prediction of peak oxygen consumption by Incremental Shuttle Walking Test in healthy women. *PloS One* **2019,** *14* (2), e0211327.
62. Berger, R., Evaluation of the 2-minute sit-up test as a measure of muscular endurance and strength. *Journal of the Association for Physical and Mental Rehabilitation* **1966,** *20* (4), 140.
63. Wells, K. F.; Dillon, E. K., The sit-and-reach. A test of back and leg flexibility. *Research Quarterly for Exercise and Sport* **1952,** *23*, 115-118.
64. DeWitt, R., A study of the sit-up Type of test as a means of measuring strength and endurance of the abdominal muscles. *Research Quarterly* **1944,** *15* (1), 60-63.

65. Mathews, D. K.; Shaw, V.; Bohnen, M., Hip flexibility of college women as related to length of body segments. *Research Quarterly* **1957,** *28* (4), 352-356.
66. Harvey, V. P.; Scott, G. D., An investigation of the curl-down test as a measure of abdominal strength. *Research Quarterly* **1967,** *38* (1), 22-27.
67. Knudson, D.; Johnston, D., Validity and reliability of a bench trunk-curl test of abdominal endurance. *Journal of Strength and Conditioning Research* **1995,** *9* (3), 165-169.
68. Clemons, J. M.; Duncan, C. A.; Blanchard, O. E.; Gatch, W. H.; Hollander, D. B.; Doucet, J. L., Relationships between the flexed-arm hang and select measures of muscular fitness. *Journal of Strength and Conditioning Research* **2004,** *18* (3), 630-636.
69. Wood, H. M.; Baumgartner, T. A., Objectivity, reliability, and validity of the bent-knee push-up for college-age women. *Meas Phys Educ Exerc Sci* **2004,** *8* (4), 203-212.
70. Clemons, J. M.; Campbell, B.; Jeansonne, C., Validity and reliability of a new test of upper body power. *Journal of Strength and Conditioning Research* **2010,** *24* (6), 1559-1565.
71. Clemons, J. M., Construct validity of a modification of the flexed arm hang test. *Journal of Strength and Conditioning Research* **2014,** *28* (12), 3523-3530.
72. Cotten, D. J., A comparison of selected trunk flexibility tests. *American Corrective Therapy Journal* **1972,** *26* (1), 24-26.
73. Jackson, A.; Langford, N. J., The criterion-related validity of the sit and reach test: replication and extension of previous findings. *Research Quarterly for Exercise and Sport* **1989,** *60* (4), 384-387.
74. Kippers, V.; Parker, A. W., Toe-touch test: A measure of its validity. *Physical Therapy* **1987,** *67* (11), 1680-1684.
75. Liemohn, W.; Sharpe, G. L.; Wasserman, J. F., Criterion related validity of the sit-and-reach test. *Journal of Strength and Conditioning Research* **1994,** *8* (2), 91-94.
76. Diener, M. H.; Golding, L. A.; Diener, D., Validity and reliability of a one-minute half sit-up test of abdominal strength and endurance. *Research in Sports Medicine* **1995,** *6* (2), 105-119.
77. Mannion, A. F.; Connolly, B.; Wood, K.; Dolan, P., The use of surface EMG power spectral analysis in the evaluation of back muscle function. *Journal of Rehabilitation Research and Development* **1997,** *34* (4), 427-39.
78. Mathiowetz, V., Comparison of Rolyan and Jamar dynamometers for measuring grip strength. *Occupational Therapy International* **2002,** *9* (3), 201-209.
79. Baumgartner, T. A.; Gaunt, S. J., Construct related validity for the Baumgartner Modified pull-up test. *Meas Phys Educ Exerc Sci* **2005,** *9* (1), 51-60.
80. López-Miñarro, P.; Vaquero-Cristóbal, R.; Muyor, J. M.; Espejo-Antúnez, L., Criterion-realted validity of sit-and-reach test as measure of hamstring extensibility in older women. *Nutricion Hospitalaria* **2015,** *32* (1), 312-7.

81. Silva, P.; Franco, J.; Gusmao, A.; Moura, J.; Teixeira-Salmela, L.; Faria, C., Trunk strength is associated with sit-to-stand performance in both stroke and healthy subjects. *European Journal of Physical and Rehabilitation Medicine* **2015,** *51* (6), 717-724.
82. Broer, M. R.; Galles, N. R. G., Importance of relationship between various body measurements in performance of the toe-touch test. *Research Quarterly* **1958,** *29* (3), 253-263.
83. Hall, G. L.; Hetzler, R. K.; Perrin, D.; Weltman, A., Relationship of timed sit-up tests to isokinetic abdominal strength. *Research Quarterly for Exercise and Sport* **1992,** *63* (1), 80-84.
84. Härkönen, R.; Harju, R.; Alaranta, H., Accuracy of the Jamar dynamometer. *Journal of Hand Therapy* **1993,** *6* (4), 259-262.
85. Sparto, P. J.; Parnianpour, M.; Reinsel, T. E.; Simon, S., Spectral and temporal responses of trunk extensor electromyography to an isometric endurance test. *Spine* **1997,** *22* (4), 418-25; discussion 425-26.
86. Knudson, D., The validity of recent curl-up tests in young adults. *Journal of Strength and Conditioning Research* **2001,** *15* (1), 81-85.
87. Youdas, J. W.; Krause, D. A.; Hollman, J. H., Validity of hamstring muscle length assessment during the sit-and-reach test using an inclinometer to measure hip joint angle. *Journal of Strength and Conditioning Research* **2008,** *22* (1), 303-309.
88. Bohannon, R. W.; Bubela, D. J.; Magasi, S. R.; Wang, Y.-C.; Gershon, R. C., Sit-to-stand test: Performance and determinants across the age-span. *Isokinetics and Exercise Science* **2010,** *18* (4), 235-240.
89. Ayala, F.; Sainz de Baranda, P.; De Ste Croix, M.; Santonja, F., Reproducibility and concurrent validity of hip joint angle test for estimating hamstring flexibility in recreationally active young me. *Journal of Strength and Conditioning Research* **2012,** *26* (9), 2372-2382.
90. Mier, C. M.; Shapiro, B. S., Sex differences in pelvic and hip flexibility in men and women matched for sit-and-reach score. *Journal of Strength and Conditioning Research* **2013,** *27* (4), 1031-1035.
91. Taylor, J. D.; Fletcher, J. P., Correlation between the 8-repetition maximum test and isokinetic dynamometry in the measurement of muscle strength of the knee extensors: A concurrent validity study. *Physiotherapy Theory and Practice* **2013,** *29* (4), 335-341.
92. Ten Hoor, G. A.; Musch, K.; Meijer, K.; Plasqui, G., Test-retest reproducibility and validity of the back-leg-chest strength measurements. *Isokinetics and Exercise Science* **2016,** *24* (3), 209-216.
93. Clemons, J., Construct Validity of Two Different Methods of Scoring and Performing Push-ups. *Journal of Strength and Conditioning Research* **2019,** *33* (11), 2971-2980.
94. Applegate, M. E.; France, C. R.; Russ, D. W.; Leitkam, S. T.; Thomas, J. S., Sorensen test performance is driven by different physiological and psychological variables in participants with and without recurrent low back pain. *Journal of Electromyography and Kinesiology* **2019,** *44*, 1-7.
95. Mannion, A. F.; Dolan, P., Electromyographic median frequency changes during isometric contraction of the back extensors to fatigue. *Spine* **1994,** *19* (11), 1223-9.

96. Kankaanpää, M.; Laaksonen, D.; Taimela, S.; Kokko, S. M.; Airaksinen, O.; Hänninen, O., Age, sex, and body mass index as determinants of back and hip extensor fatigue in the isometric Sørensen back endurance test. *Archives of Physical Medicine and Rehabilitation* **1998,** *79* (9), 1069-75.
97. Shechtman, O.; Gestewitz, L.; Kimble, C., Reliability and validity of the DynEx dynamometer. *Journal of Hand Therapy* **2005,** *18* (3), 339-347.
98. Coorevits, P.; Danneels, L.; Cambier, D.; Ramon, H.; Vanderstraeten, G., Assessment of the validity of the Biering-Sørensen test for measuring back muscle fatigue based on EMG median frequency characteristics of back and hip muscles. *Journal of Electromyography and Kinesiology* **2008,** *18* (6), 997-1005.
99. Bui, H. T.; Farinas, M.-I.; Fortin, A.-M.; Comtois, A.-S.; Leone, M., Comparison and analysis of three different methods to evaluate vertical jump height. *Clinical Physiology and Functional Imaging* **2015,** *35* (3), 203-209.
100. De Blaiser, C.; De Ridder, R.; Willems, T.; Danneels, L.; Roosen, P., Reliability and validity of trunk flexor and trunk extensor strength measurements using handheld dynamometry in a healthy athletic population. *Physical Therapy in Sport* **2018,** *34*, 180-186.
101. Kawano, M. M.; Ambar, G.; Oliveira, B. I. R.; Boer, M. C.; Cardoso, A. P. R. G.; Cardoso, J. R., Influence of the gastrocnemius muscle on the sit-and-reach test assessed by angular kinematic analysis. *Braz J Phys Ther* **2010,** *14* (1), 10-15.
102. España-Romero, V.; Ortega, F. B.; Vicente-Rodríguez, G.; Artero, E. G.; Rey, J. P.; Ruiz, J. R., Elbow position affects handgrip strength in adolescents: validity and reliability of Jamar, DynEx, and TKK dynamometers. *Journal of Strength and Conditioning Research* **2010,** *24* (1), 272-277.
103. Cadenas-Sanchez, C.; Sanchez-Delgado, G.; Martinez-Tellez, B.; Mora-Gonzalez, J.; Löf, M.; España-Romero, V.; Ruiz, J. R.; Ortega, F. B., Reliability and validity of different models of TKK hand dynamometers. *American Journal of Occupational Therapy* **2016,** *70* (4), 7004300010.
104. Kolimechkov, S.; Castro-Piñero, J.; Petrov, A.; Alexandrova, A., The effect of elbow position on the handgrip strength test in children: validity and reliability of TKK 5101 and DynX dynamometers. *Pedagogy Phys Cult Sports* **2020,** *24* (5), 240-247.
105. Miyamoto, K.; Takebayashi, H.; Takimoto, K.; Miyamoto, S.; Inoue, Y.; Takuma, Y.; Okabe, T.; Morioka, S.; Yagi, F., The criterion-related validity of the ten step test compared with motor reaction time. *J Phys Ther Sci* **2008,** *20* (4), 261-265.
106. Miyamoto, K.; Takebayashi, H.; Takimoto, K.; Miyamoto, S.; Morioka, S.; Yagi, F., A new simple performance test focused on agility in elderly people: The Ten Step Test. *Gerontology* **2008,** *54* (6), 365-372.
107. Wells, K. F.; Dillon, E. K., The sit and reach—a test of back and leg flexibility. *Research Quarterly for Exercise and Sport* **1952,** *23* (1), 115-118.

Supplementary material 1: Search strategy terms

MEDLINE (via PubMed): all searches combined

(((((((((((((((((Adult[Mesh:NoExp] OR "Middle Aged"[Mesh]) AND ("criterion validity" [All Fields] OR “criterion-related validity”[All Fields] OR validity[All Fields] OR validation[All Fields] OR cross-validation [All Fields]) AND (“Physical fitness"[Mesh])))) OR (((Adult[Mesh:NoExp] OR "Middle Aged"[Mesh]) AND ("criterion validity"[All Fields] OR “criterion-related validity”[All Fields] OR validity[All Fields] OR validation[All Fields] OR cross-validation[All Fields]) AND ("Muscle Strength"[Mesh] OR "Muscle strength dynamometer"[Mesh])))) OR (((Adult[Mesh:NoExp] OR "Middle Aged"[Mesh]) AND ("criterion validity"[All Fields] OR “criterion-related validity”[All Fields] OR validity[All Fields] OR validation [All Fields] OR cross-validation [All Fields]) AND (“Range of motion, articular”[Mesh Major Topic])))) OR (((Adult[Mesh:NoExp] OR "Middle Aged"[Mesh]) AND ("criterion validity" [All Fields] OR “criterion-related validity” [All Fields] OR validity [All Fields] OR validation [All Fields] OR cross-validation [All Fields]) AND ("Postural Balance"[MeSH])))) OR (((Adult[Mesh:NoExp] OR "Middle Aged"[Mesh]) AND ("criterion validity" [All Fields] OR “criterion-related validity” [All Fields] OR validity [All Fields] OR validation [All Fields] OR cross-validation [All Fields]) AND (“Physical Endurance"[MeSH])))) OR (((Adult[Mesh:NoExp] OR "Middle Aged"[Mesh]) AND ("criterion validity" [All Fields] OR “criterion-related validity” [All Fields] OR validity [All Fields] OR validation [All Fields] OR cross-validation [All Fields]) AND (“Cardiorespiratory fitness”[All Fields] OR “Cardiovascular fitness”[All Fields] OR “Aerobic fitness”[All Fields] OR “Aerobic capacity”[All Fields] OR “Maximal oxygen consumption”[All Fields] OR “VO2max”[All Fields])))) OR (((Adult[Mesh:NoExp] OR "Middle Aged"[Mesh]) AND ("criterion validity" [All Fields] OR “criterion-related

validity” [All Fields] OR validity [All Fields] OR validation [All Fields] OR cross-validation [All Fields]) AND ("Motor fitness"[All Fields])))) OR (((Adult[Mesh:NoExp] OR "Middle Aged"[Mesh]) AND ("criterion validity" [All Fields] OR “criterion-related validity” [All Fields] OR validity [All Fields] OR validation [All Fields] OR cross-validation [All Fields]) AND ("Running Speed"[All Fields])))) OR (((Adult[Mesh:NoExp] OR "Middle Aged"[Mesh]) AND ("criterion validity" [All Fields] OR “criterion-related validity” [All Fields] OR validity [All Fields] OR validation [All Fields] OR cross-validation [All Fields]) AND (Agility [All Fields])))) OR (((Adult[Mesh:NoExp] OR "Middle Aged"[Mesh]) AND ("criterion validity"[All Fields] OR “criterion-related validity”[All Fields] OR validity[All Fields] OR validation [All Fields] OR cross-validation [All Fields]) AND (EUROFIT[All Fields])))) OR (((Adult[Mesh:NoExp] OR "Middle Aged"[Mesh]) AND ("criterion validity"[All Fields] OR “criterion-related validity”[All Fields] OR validity[All Fields] OR validation[All Fields] OR cross-validation [All Fields]) AND (HRFT-UKK[All Fields])))) OR (((Adult[Mesh:NoExp] OR "Middle Aged"[Mesh]) AND ("criterion validity" [All Fields] OR “criterion-related validity”[All Fields] OR validity[All Fields] OR validation[All Fields] OR cross-validation[All Fields]) AND (ALPHA-FIT[All Fields])))) OR (((Adult[Mesh:NoExp] OR "Middle Aged"[Mesh]) AND ("criterion validity"[All Fields] OR “criterion-related validity”[All Fields] OR validity[All Fields] OR validation[All Fields] OR cross-validation[All Fields]) AND (AFISAL-INEFC[All Fields])))) OR (((Adult[Mesh:NoExp] OR "Middle Aged"[Mesh]) AND ("criterion validity"[All Fields] OR “criterion-related validity”[All Fields] OR validity[All Fields] OR validation[All Fields] OR cross-validation[All Fields]) AND (CAHPER[All Fields])))) OR (((Adult[Mesh:NoExp] OR "Middle Aged"[Mesh]) AND ("criterion validity" [All Fields]

OR “criterion-related validity”[All Fields] OR validity[All Fields] OR validation[All Fields] OR cross-validation[All Fields]) AND (CPAFLA[All Fields])))

Filters activated: Publication date from 1990/01/01 to 2020/07/10, Humans, English, Spanish

Web of Sciences: all searches combined

(adult* OR "middle aged") AND ("criterion validity" OR "criterion-related validity" OR validity OR validation OR "cross-validation") AND ("Physical fitness" OR "Physical Conditioning") OR (adult* OR "middle aged") AND ("criterion validity" OR "criterion-related validity" OR validity OR validation OR "cross-validation") AND ("Muscle strength" OR "Muscular strength" OR dynamometer) OR (adult* OR "middle aged") AND ("criterion validity" OR "criterion-related validity" OR validity OR validation OR "cross-validation") AND (“Joint Range of Motion” OR “Joint flexibility” OR “Range of motion”) AND (Test) OR (adult* OR "middle aged") AND ("criterion validity" OR "criterion-related validity" OR validity OR validation OR "cross-validation") AND (“Musculoskeletal Equilibrium” OR “Postural Balance” OR “Postural Equilibrium”) OR (adult* OR "middle aged") AND ("criterion validity" OR "criterion-related validity" OR validity OR validation OR "cross-validation") AND (“Cardiorespiratory fitness” OR “Cardiovascular fitness” OR “Aerobic fitness” OR “Aerobic fitness” OR “Aerobic capacity” OR “Maximal oxygen consumption” OR “VO2max” OR "Physical Endurance") OR (adult* OR "middle aged") AND ("criterion validity" OR "criterion-related validity" OR validity OR validation OR "cross-validation") AND (“Running Speed” OR “Agility” OR "Motor fitness") OR (adult* OR "middle aged") AND ("criterion validity" OR "criterion-related validity" OR validity OR validation OR "cross-validation") AND (EUROFIT) OR (adult* OR "middle aged") AND ("criterion validity"

OR "criterion-related validity" OR validity OR validation OR "cross-validation") AND (HRFT-UKK) OR (adult* OR "middle aged") AND ("criterion validity" OR "criterion-related validity" OR validity OR validation OR "cross-validation") AND (ALPHA-FIT) OR (adult* OR "middle aged") AND ("criterion validity" OR "criterion-related validity" OR validity OR validation OR "cross-validation") AND (AFISAL-INEFC) OR (adult* OR "middle aged") AND ("criterion validity" OR "criterion-related validity" OR validity OR validation OR "cross-validation") AND (CAHPER) OR (adult* OR "middle aged") AND ("criterion validity" OR "criterion-related validity" OR validity OR validation OR "cross-validation") AND (CPAFLA)

Terms used in the search strategy in MEDLINE (via Pubmed)

Search criteria 1	Search criteria 2	MeSH Entry Terms for Criteria 2	Search criteria 3
	Physical fitness (MeSH)	Fitness, Physical	
	Muscle Strength (MeSH)	Strength, Muscle	
	Muscle strength dynamometer (MeSH)	Dynamometer, Muscle Strength<br>Dynamometers, Muscle Strength<br>Muscle<br>Strength Dynamometers	
	Range of motion, articular (MeSH Major Topic*)	Joint Range of Motion<br>Joint Flexibility<br>Flexibility, Joint<br>Range of Motion<br>Passive Range of Motion	
("Adult"[Mesh:NoExp] OR "Middle	Postural Balance (MeSH)	Musculoskeletal Equilibrium<br>Equilibrium, Musculoskeletal<br>Postural Equilibrium<br>Equilibrium, Postural<br>Balance, Postural	(“validity" OR "criterion-related validity" OR validity

Aged"[Mesh])	Cardiorespiratory fitness	-	OR validation OR "cross-validation")
	Cardiovascular fitness	-	
	Aerobic fitness	-	
	Aerobic capacity	-	
	Maximal oxygen consumption	-	
	VO_2max	-	
	Running Speed	-	
	Agility	-	
	Physical Endurance	Endurance, Physical<br>Endurances, Physical<br>Physical Endurances	
	Motor fitness	-	
	EUROFIT<br>HRFT-UKK<br>ALPHA-FIT<br>AFISAL-INEFC<br>CAHPER<br>CPAFLA	-<br>-<br>-<br>-<br>-<br>-	

Supplementary material 2: Results of low and very low quality studies

Criterion-related validity

Cardiorespiratory fitness

Distance and time-based run/walk tests

One very low quality study suggested that the University Montreal test [6] was valid for estimating $VO2_{max}$ (r=0.96, P<.05) (see supplementary table S3). Low quality studies reported that the 400-m [15] (ρ= -0.56, P<.001) and 6-minute walk [17] tests (r=0.85, P<.05) had high validity; the 1500-m fast walk test [18] had moderate validity (r=0.52, P<.01); and the 2.4-km run [10], 1-mile walk [10] and 4-minute run [11] tests had low validity (r=0.42, P=0.13; r=0.18, P=0.52; mean difference=1.6±3.6 ml/kg/min, P<.05, respectively) to assess $VO2_{max}$.

20-m shuttle run test

One low quality study [13] investigated the criterion-related validity of the 20-m shuttle run test, reporting that this test was valid to assess cardiorespiratory fitness (r=0.90, P<.001) (see supplementary table S3).

Step tests

Three low quality studies revealed that the 6-minute step [12, 19] and 3-minute single step [8] tests, were valid (r=0.72-0.92, both P<.05) (see supplementary table S3). However, 2-level step [9], the USO step [7], modified Harvard step [16], and Danish step [21] tests reported low validity (r=0.47-0.66, all P<.01).

Muscular strength

Maximal isometric strength

Two and 1 low quality studies observed that the Jamar [78, 84] and Rolyan [78] dynamometer, respectively, were valid (r =0.99, both P<.05). Moreover, one very low

quality study concluded that the modified flex-hang arm test [71] was valid to asses upper body relative isometric strength (r=0.88, p< 0.001).

Endurance strength

Thirteen low quality studies examined the criterion-related validity of endurance strength (see supplementary table S3). The Baumgartner modified pull-up test [79] turned out to be valid to assess upper body endurance strength (r=0.60-0.85, P<.05), while the back-leg-chest test [92] had moderate-high validity (r=0.56-0.81, all P<.01). The standard push-up test [93] had moderate validity to assess relative upper body endurance strength (r=0.7, P<.001) but not the hand-release push-up test [93] (rho=0.39, P<.05). Three studies reported that the curl-up test or its modifications [76, 83, 86] were not valid to assess trunk endurance strength (r=0.32-0.68, P<.05). While 3 studies [77, 85, 94] showed that the Biering-Sørensen test had moderate (r=0.75, P<.001) to high (r=0.92-0.94, P<.05) validity to assess trunk endurance strength. Four studies [81, 88, 91] found inconclusive results when analysing the validity of the sit-to-stand test different versions to assess lower body endurance strength.

Two very low quality studies observed that the flexed-arm hang, [68] and bent-knee push-up and revised push-up [69] tests had acceptable validity to assess upper body endurance strength (r=0.67-0.72, P<.05); and 4 very low quality studies concluded that different versions of the curl-up test [62, 64, 66, 67] were not valid to asses trunk endurance strength (r=0.04-0.51, P<.05).

Explosive strength

One very low quality study reported that the medicine ball put test [70] is valid to asses upper body explosive strength in female and male (r=0.86 and 0.79, respectively, both P<.01).

Flexibility

Six low quality studies determined that the sit-and-reach test [72, 73, 75, 80, 87, 90] and its different versions [72, 75] had low-moderate validity to assess low back and hamstring flexibility (r=0.12-0.82, P<.05). However, the toe-touch test [74, 82] showed high validity to assess hamstring flexibility (r=0.81-0.85, both P<.05). One low quality studies reported that the horizontal and vertical hip joint angle tests [89] had moderate validity (r=0.79, P<.01).

Two studies classified as very low quality observed that the sit-and-reach [107] (r=0.90, P<.05) and the adapted Kraus-Weber [65] tests (r=0.74-0.82, P<.05) were valid to assess hip and hamstring and low back flexibility, respectively.

Motor fitness

Two low quality studies found that the ten step test [105, 106] had moderate validity to estimate agility (r=0.35-0.68, P<.01). Another low quality study concluded that the Romberg test [9] was not valid to assess the balance (r= -0.18-0.23, P>.05).

REFERENCES

1. Leger, L.; Boucher, R., An indirect continuous running multistage field test: the Universite de Montreal track test. *Can. J. Appl. Sport. Sci* **1980,** *5*, 77-84.
2. Gabriel, K. K. P.; Rankin, R. L.; Lee, C.; Charlton, M. E.; Swan, P. D.; Ainsworth, B. E.; Pettee Gabriel, K. K.; Rankin, R. L.; Lee, C.; Charlton, M. E.; Swan, P. D.; Ainsworth, B. E., Test-retest reliability and validity of the 400-meter walk test in healthy, middle-aged women. *J Phys Act Health* **2010,** *7* (5), 649-657.
3. Burr, J. F.; Bredin, S. S.; Faktor, M. D.; Warburton, D. E., The 6-minute walk test as a predictor of objectively measured aerobic fitness in healthy working-aged adults. *Physician and Sportsmedicine* **2011,** *39* (2), 133-9.
4. Mikawa, K.; Senjyu, H., Development of a field test for evaluating aerobic fitness in middle-aged adults: Validity of a 15-m Incremental Shuttle Walk and Run Test. *Journal of Sports Science & Medicine* **2011,** *10* (4), 712-717.
5. Azmi, S. H.; Sulaiman, N., Validity of selected cardiovascular field-based test among Malaysian healthy female adult. *J Fundam Appl Sci* **2017,** *9*, 1227-1235.
6. McGawley, K., The Reliability and Validity of a Four-Minute Running Time-Trial in Assessing VO2max and Performance. *Frontiers in Physiology* **2017,** *8*, 270.
7. Leger, L. A.; Mercier, D.; Gadoury, C.; Lambert, J., The multistage 20 metre shuttle run test for aerobic fitness. *JOURNAL OF SPORTS SCIENCES* **1988,** *6* (2), 93-101.

8. Giacomantonio, N.; Morrison, P.; Rasmussen, R.; MacKay-Lyons, M. J., Reliability and validity of the 6-minutestep test for clinical assessment of cardiorespiratory fitness in people at risk of cardiovascular disease. *Journal of Strength and Conditioning Research* **2020,** *34* (5), 1376-1382; Arcuri, J. F.; Borghi-Silva, A.; Labadessa, I. G.; Sentanin, A. C.; Candolo, C.; Pires Di Lorenzo, V. A., Validity and reliability of the 6-minute step test in healthy individuals: a cross-sectional study. *Clinical Journal of Sport Medicine* **2016,** *26* (1), 69-75.
9. Siconolfi, S. F.; Garber, C. E.; Lasater, T. M.; Carleton, R. A., A simple, valid step test for estimating maximal oxygen uptake in epidemiologic studies. *American Journal of Epidemiology* **1985,** *121* (3), 382-390.
10. Ritchie, C.; Trost, S. G.; Brown, W.; Armit, C., Reliability and validity of physical fitness field tests for adults aged 55 to 70 years. *Journal of Science and Medicine in Sport* **2005,** *8* (1), 61-70.
11. Santa Maria, D.; Kinnear, G. R.; Kearney, J. T.; Martin, T. P., The objectivity, reliability, and validity of the OSU step test for college males. *Research Quarterly* **1976,** *47* (3), 445-452.
12. Hansen, D.; Jacobs, N.; Bex, S.; D'Haene, G.; Dendale, P.; Claes, N., Are fixed-rate step tests medically safe for assessing physical fitness? *European Journal of Applied Physiology* **2011,** *111* (10), 2593-2599.
13. Lerche, L.; Olsen, A.; Petersen, K. E. N.; Rostgaard-Hansen, A. L.; Dragsted, L. O.; Nordsborg, N. B.; Tjonneland, A.; Halkjaer, J., Validity of physical activity and cardiorespiratory fitness in the Danish cohort "Diet, Cancer and Health-Next Generations". *Scandinavian Journal of Medicine & Science in Sports* **2017,** *27* (12), 1864-1872.
14. Mathiowetz, V., Comparison of Rolyan and Jamar dynamometers for measuring grip strength. *Occupational Therapy International* **2002,** *9* (3), 201-209.
15. Härkönen, R.; Harju, R.; Alaranta, H., Accuracy of the Jamar dynamometer. *Journal of Hand Therapy* **1993,** *6* (4), 259-262.
16. Clemons, J. M., Construct validity of a modification of the flexed arm hang test. *Journal of Strength and Conditioning Research* **2014,** *28* (12), 3523-3530.
17. Baumgartner, T. A.; Gaunt, S. J., Construct related validity for the Baumgartner Modified pull-up test. *Meas Phys Educ Exerc Sci* **2005,** *9* (1), 51-60.
18. Ten Hoor, G. A.; Musch, K.; Meijer, K.; Plasqui, G., Test-retest reproducibility and validity of the back-leg-chest strength measurements. *Isokinetics and Exercise Science* **2016,** *24* (3), 209-216.
19. Clemons, J., Construct Validity of Two Different Methods of Scoring and Performing Push-ups. *Journal of Strength and Conditioning Research* **2019,** *33* (11), 2971-2980.
20. Diener, M. H.; Golding, L. A.; Diener, D., Validity and reliability of a one-minute half sit-up test of abdominal strength and endurance. *Research in Sports Medicine* **1995,** *6* (2), 105-119; Knudson, D., The validity of recent curl-up tests in young adults. *Journal of Strength and Conditioning Research* **2001,** *15* (1), 81-85; Hall, G. L.; Hetzler, R. K.; Perrin, D.; Weltman, A., Relationship of timed sit-up tests to isokinetic abdominal strength. *Research Quarterly for Exercise and Sport* **1992,** *63* (1), 80-84.
21. Mannion, A. F.; Connolly, B.; Wood, K.; Dolan, P., The use of surface EMG power spectral analysis in the evaluation of back muscle function. *Journal of Rehabilitation Research and Development* **1997,** *34* (4), 427-39; Sparto, P. J.; Parnianpour, M.; Reinsel, T. E.; Simon, S., Spectral and temporal responses of trunk extensor electromyography to an isometric endurance test. *Spine* **1997,** *22* (4), 418-25;

discussion 425-26; Applegate, M. E.; France, C. R.; Russ, D. W.; Leitkam, S. T.; Thomas, J. S., Sorensen test performance is driven by different physiological and psychological variables in participants with and without recurrent low back pain. *Journal of Electromyography and Kinesiology* **2019,** *44*, 1-7.
22. Bohannon, R. W.; Bubela, D. J.; Magasi, S. R.; Wang, Y.-C.; Gershon, R. C., Sit-to-stand test: Performance and determinants across the age-span. *Isokinetics and Exercise Science* **2010,** *18* (4), 235-240; Silva, P.; Franco, J.; Gusmao, A.; Moura, J.; Teixeira-Salmela, L.; Faria, C., Trunk strength is associated with sit-to-stand performance in both stroke and healthy subjects. *European Journal of Physical and Rehabilitation Medicine* **2015,** *51* (6), 717-724; Taylor, J. D.; Fletcher, J. P., Correlation between the 8-repetition maximum test and isokinetic dynamometry in the measurement of muscle strength of the knee extensors: A concurrent validity study. *Physiotherapy Theory and Practice* **2013,** *29* (4), 335-341.
23. Clemons, J. M.; Duncan, C. A.; Blanchard, O. E.; Gatch, W. H.; Hollander, D. B.; Doucet, J. L., Relationships between the flexed-arm hang and select measures of muscular fitness. *Journal of Strength and Conditioning Research* **2004,** *18* (3), 630-636.
24. Wood, H. M.; Baumgartner, T. A., Objectivity, reliability, and validity of the bent-knee push-up for college-age women. *Meas Phys Educ Exerc Sci* **2004,** *8* (4), 203-212.
25. Harvey, V. P.; Scott, G. D., An investigation of the curl-down test as a measure of abdominal strength. *Research Quarterly* **1967,** *38* (1), 22-27; Berger, R., Evaluation of the 2-minute sit-up test as a measure of muscular endurance and strength. *Journal of the Association for Physical and Mental Rehabilitation* **1966,** *20* (4), 140; Knudson, D.; Johnston, D., Validity and reliability of a bench trunk-curl test of abdominal endurance. *Journal of Strength and Conditioning Research* **1995,** *9* (3), 165-169; DeWitt, R., A study of the sit-up Type of test as a means of measuring strength and endurance of the abdominal muscles. *Research Quarterly* **1944,** *15* (1), 60-63.
26. Clemons, J. M.; Campbell, B.; Jeansonne, C., Validity and reliability of a new test of upper body power. *Journal of Strength and Conditioning Research* **2010,** *24* (6), 1559-1565.
27. Jackson, A.; Langford, N. J., The criterion-related validity of the sit and reach test: replication and extension of previous findings. *Research Quarterly for Exercise and Sport* **1989,** *60* (4), 384-387; Youdas, J. W.; Krause, D. A.; Hollman, J. H., Validity of hamstring muscle length assessment during the sit-and-reach test using an inclinometer to measure hip joint angle. *Journal of Strength and Conditioning Research* **2008,** *22* (1), 303-309; Mier, C. M.; Shapiro, B. S., Sex differences in pelvic and hip flexibility in men and women matched for sit-and-reach score. *Journal of Strength and Conditioning Research* **2013,** *27* (4), 1031-1035; López-Miñarro, P.; Vaquero-Cristóbal, R.; Muyor, J. M.; Espejo-Antúnez, L., Criterion-realted validity of sit-and-reach test as measure of hamstring extensibility in older women. *Nutricion Hospitalaria* **2015,** *32* (1), 312-7.
28. Liemohn, W.; Sharpe, G. L.; Wasserman, J. F., Criterion related validity of the sit-and-reach test. *Journal of Strength and Conditioning Research* **1994,** *8* (2), 91-94; Cotten, D. J., A comparison of selected trunk flexibility tests. *American Corrective Therapy Journal* **1972,** *26* (1), 24-26.
29. Broer, M. R.; Galles, N. R. G., Importance of relationship between various body measurements in performance of the toe-touch test. *Research Quarterly* **1958,** *29* (3), 253-263; Kippers, V.; Parker, A. W., Toe-touch test: A measure of its validity. *Physical Therapy* **1987,** *67* (11), 1680-1684.

30. Ayala, F.; Sainz de Baranda, P.; De Ste Croix, M.; Santonja, F., Reproducibility and concurrent validity of hip joint angle test for estimating hamstring flexibility in recreationally active young me. *Journal of Strength and Conditioning Research* **2012,** *26* (9), 2372-2382.
31. Wells, K. F.; Dillon, E. K., The sit and reach—a test of back and leg flexibility. *Research Quarterly for Exercise and Sport* **1952,** *23* (1), 115-118.
32. Mathews, D. K.; Shaw, V.; Bohnen, M., Hip flexibility of college women as related to length of body segments. *Research Quarterly* **1957,** *28* (4), 352-356.
33. Miyamoto, K.; Takebayashi, H.; Takimoto, K.; Miyamoto, S.; Inoue, Y.; Takuma, Y.; Okabe, T.; Morioka, S.; Yagi, F., The criterion-related validity of the ten step test compared with motor reaction time. *J Phys Ther Sci* **2008,** *20* (4), 261-265; Miyamoto, K.; Takebayashi, H.; Takimoto, K.; Miyamoto, S.; Morioka, S.; Yagi, F., A new simple performance test focused on agility in elderly people: The Ten Step Test. *Gerontology* **2008,** *54* (6), 365-372.

Supplementary material 3. References of high quality studies included in the systematic review.

Mayorga-Vega D, Aguilar-Soto P, Viciana J Criterion-related validity of the 20-m shuttle run test for estimating cardiorespiratory fitness: a meta-analysis. *J Sports Sci Med* 2015;14:536-547.

Mayorga-Vega D, Bocanegra-Parrilla R, Ornelas M *et al.*Criterion-related validity of the distance- and time-based walk/run field tests for estimating cardiorespiratory fitness: a systematic review and Meta-analysis. *PLoS One* 2016;11:e0151671-e0151671.

Bennett H, Parfitt G, Davison K *et al.* Validity of submaximal step tests to estimate maximal oxygen uptake in healthy adults. *Sports Med* 2016;46:737-750.

Moher D, Liberati A, Tetzlaff J *et al.* Preferred reporting items for systematic reviews and meta-analyses: the PRISMA statement. *PLoS Med* 2009;6:e1000097.

Shea BJ, Hamel C, Wells GA *et al.* AMSTAR is a reliable and valid measurement tool to assess the methodological quality of systematic reviews. *J Clin Epidemiol* 2009;62:1013-1020.

Oja P, Laukkanen R, Pasanen M *et al.* A 2-km walking test for assessing the cardiorespiratory fitness of healthy adults. *Int J Sports Med* 1991;12:356-362.

Laukkanen R, Oja P, Pasanen M *et al.* Validity of a two kilometre walking test for estimating maximal aerobic power in overweight adults. *Int J Obes Relat Metab Disord* 1992;16:263-268.

Laukkanen RMT, Oja P, Pasanen ME *et al.*Criterion validitv of a two-kilometer walking test for predicting the maximal oxygen uptake of moderately to highly active middle-aged adults. *Scand J Med Sci Sports* 1993: 267-272.

Laukkanen RMT, Kukkonen-Harjula TK, Oja P *et al.* Prediction of change in maximal aerobic power by the 2-km walk test after walking training in middle-aged adults. *Int J Sports Med* 2000;21:113-116.

Larsen GE, George JD, Alexander JL *et al.* Prediction of maximum oxygen consumption from walking, jogging, or running. *Res Q Exerc Sport* 2002;73:66-72.

McNaughton L, Hall P, Cooley D Validation of several methods of estimating maximal oxygen uptake in young men. *Percept Mot Skills* 1998;87:575-584.

Kline C, Porcari JP, Hintermeister R *et al.* Estimation of from a one-mile track walk, gender, age and body weight. *Med Sports Exerc* 1987;19:253-259.

George JD, Fellingham GW, Fisher AG A modified version of the Rockport Fitness Walking Test for college men and women. *Res Q Exerc Sport* 1998;69:205-209.

Lunt H, Roiz De Sa D, Roiz De Sa J *et al.*Validation of one-mile walk equations for the estimation of aerobic fitness in British military personnel under the age of 40 years. *Mil Med* 2013;178:753-759.

Greenhalgh HA, George JD, Hager RL Cross-validation of a quarter-mile walk test using two VO2 max regression models. *Meas Phys Educ Exerc Sci* 2001;5:139-151.

Dolgener FA, Hensley LD, Marsh JJ *et al.* Validation of the Rockport Fitness Walking Test in college males and females. *Res Q Exerc Sport* 1994;65:152-158.

Seneli RM, Ebersole KT, O'Connor KM *et al.* Estimated VO2max from the Rockport Walk Test on a Nonmotorized Curved Treadmill. *J Strength Cond Res* 2013;27:3495-3505.

George JD, Vehrs PR, Allsen PE *et al.* Development of a submaximal treadmill jogging test for fit college-aged individuals. *Med Sci Sports Exerc* 1993;25:643-647.

Cao Z-B, Miyatake N, Aoyama T *et al.* Prediction of maximal oxygen uptake from a 3-minute walk based on gender, age, and body composition. *J Phys Act Health* 2013;10:280-287.

Di Thommazo-Luporini L, Pinheiro Carvalho L, Luporini R *et al.* The six-minute step test as a predictor of cardiorespiratory fitness in obese women. *Eur J Phys Rehabil Med* 2015;51:793-802.

Di Thommazo-Luporini L, Carvalho LP, Luporini RL *et al.* Are cardiovascular and metabolic responses to field walking tests interchangeable and obesity-dependent? *Disabil Rehabil* 2016;38:1820-1829.

Manttari A, Suni J, Sievanen H *et al.*Six-minute walk test: a tool for predicting maximal aerobic power (VO2 max) in healthy adults. *Clin Physiol Funct Imaging* 2018.

Bonet JB, Magalhaes J, Viscor G *et al.* A field tool for the aerobic power evaluation of middle-aged female recreational runners. *Women Health* 2020.

Cooper SM, Baker JS, Tong RJ *et al.* The repeatability and criterion related validity of the 20 m multistage fitness test as a predictor of maximal oxygen uptake in active young men. *Br J Sports Med* 2005;39:e19.

Flouris AD, Koutedakis Y, Nevill A *et al.* Enhancing specificity in proxy-design for the assessment of bioenergetics. *J Sci Med Sport* 2004;7:197-204.

Flouris AD, Metsios GS, Koutedakis Y Enhancing the efficacy of the 20 m multistage shuttle run test. *Br J Sports Med* 2005;39:166-170.

Metsios GS, Flouris AD, Koutedakis Y *et al.* Criterion-related validity and test-retest reliability of the 20m square shuttle test. *J Sci Med Sport* 2008;11:214-217.

Kim J, Jung SH, Cho HC Validity and Reliability of Shuttle-Run Test in Korean Adults. *Int J Sports Med* 2011;32:580-585.

Jurio-Iriarte B, Gorostegi-Anduaga I, Rodrigo Aispuru G *et al.* Association between Modified Shuttle Walk Test and cardiorespiratory fitness in overweight/obese adults with primary hypertension: EXERDIET-HTA study. *J Am Soc Hypertens* 2017;11:186-195.

Jurio-Iriarte B, Brubaker PH, Gorostegi-Anduaga I *et al.* Validity of the modified shuttle walk test to assess cardiorespiratory fitness after exercise intervention in overweight/obese adults with primary hypertension. *Clin Exp Hypertens* 2018:1-6.

Lima LP, Leite HR, Matos MA *et al.*Cardiorespiratory fitness assessment and prediction of peak oxygen consumption by Incremental Shuttle Walking Test in healthy women. *PLoS One* 2019;14:e0211327.

Aadahl M, Zacho M, Linneberg A *et al.*Comparison of the Danish step test and the watt-max test for estimation of maximal oxygen uptake: the Health2008 study. *Eur J Prev Cardiol* 2013;20:1088-1094.

Kumar SK, Khare P, Jaryal AK *et al.* Validity of heart rate based nomogram fors estimation of maximum oxygen uptake in Indian population. *Indian J Physiol Pharmacol* 2012;56:279-283.

Ricci PA, Cabiddu R, Jürgensen SP *et al.*Validation of the two-minute step test in obese with comorbibities and morbidly obese patients. *Braz J Med Biol Res* 2019;52:e8402.

Weller IM, Thomas SG, Cox MH *et al.* A study to validate the Canadian Aerobic Fitness Test. *Can J Public Health* 1992;83:120-124.

Carvalho LP, Di Thommazo-Luporini L, Aubertin-Leheudre M *et al.* Prediction of cardiorespiratory fitness by the six-minute step test and its association with muscle strength and power in sedentary obese and lean young women: a cross-sectional study. *PLoS One* 2015;10:e0145960.

Teren A, Zachariae S, Beutner F *et al.* Incremental value of veterans specific activity questionnaire and the ymca-step test for the assessment of cardiorespiratory fitness in population-based studies. *Eur J Prev Cardiol* 2016;23:1221-1227.

Beutner F, Ubrich R, Zachariae S *et al.* Validation of a brief step-test protocol for estimation of peak oxygen uptake. *Eur J Prev Cardiol* 2015;22:503-512.

Lee O, Lee S, Kang M *et al.* Prediction of maximal oxygen consumption using the Young Men's Christian Association-step test in Korean adults. *Eur J Appl Physiol* 2019;119:1245-1252.

Hong SH, Yang HI, Kim DI *et al.* Validation of submaximal step tests and the 6-min walk test for predicting maximal oxygen consumption in young and healthy participants. *Int J Environ Res Public Health* 2019;16.

Kieu NTV, Jung SJ, Shin SW *et al.* The validity of the YMCA 3-minute step test for estimating maximal oxygen uptake in healthy Korean and Vietnamese adults. *J Lifestyle Med* 2020;10:21-29.

Hansen D, Jacobs N, Thijs H *et al.* Validation of a single-stage fixed-rate step test for the prediction of maximal oxygen uptake in healthy adults. *Clin Physiol Funct Imaging* 2016;36:401-406.

Espana-Romero V, Artero EG, Santaliestra-Pasias AM *et al.* Hand span influences optimal grip span in boys and girls aged 6 to 12 years. *J Hand Surg [Am]* 2008;33:378-384.

Cadenas-Sanchez C, Sanchez-Delgado G, Martinez-Tellez B *et al.*Reliability and validity of different models of TKK hand dynamometers. *Am J Occup Ther* 2016;70:7004300010.

Kolimechkov S, Castro-Piñero J, Petrov A *et al.* The effect of elbow position on the handgrip strength test in children: validity and reliability of TKK 5101 and DynX dynamometers. *Pedagogy Phys Cult Sports* 2020;24:240-247.

Shechtman O, Gestewitz L, Kimble C Reliability and validity of the DynEx dynamometer. *J Hand Ther* 2005;18:339-347.

Mannion AF, Dolan P Electromyographic median frequency changes during isometric contraction of the back extensors to fatigue. *Spine (Phila Pa 1976)* 1994;19:1223-1229.

Coorevits P, Danneels L, Cambier D *et al.* Assessment of the validity of the Biering-Sørensen test for measuring back muscle fatigue based on EMG median frequency characteristics of back and hip muscles. *J Electromyogr Kinesiol* 2008;18:997-1005.

Kankaanpää M, Laaksonen D, Taimela S *et al.* Age, sex, and body mass index as determinants of back and hip extensor fatigue in the isometric Sørensen back endurance test. *Arch Phys Med Rehabil* 1998;79:1069-1075.

De Blaiser C, De Ridder R, Willems T *et al.* Reliability and validity of trunk flexor and trunk extensor strength measurements using handheld dynamometry in a healthy athletic population. *Phys Ther Sport* 2018;34:180-186.

Bui HT, Farinas M-I, Fortin A-M *et al.* Comparison and analysis of three different methods to evaluate vertical jump height. *Clin Physiol Funct Imaging* 2015;35:203-209.

Kawano MM, Ambar G, Oliveira BIR *et al.* Influence of the gastrocnemius muscle on the sit-and-reach test assessed by angular kinematic analysis. *Braz J Phys Ther* 2010;14:10-15.
